# Turning Vice into Virtue: Using Batch-Effects to Detect Errors in Large Genomic Data Sets

**DOI:** 10.1093/gbe/evy199

**Published:** 2018-09-10

**Authors:** Fabrizio Mafessoni, Rashmi B Prasad, Leif Groop, Ola Hansson, Kay Prüfer

**Affiliations:** 1 Department of Evolutionary Genetics, Max Planck Institute for Evolutionary Anthropology, Leipzig, Germany; 2 Department of Clinical Sciences, Diabetes and Endocrinology, Lund University Diabetes Center, Malmö, Sweden; 3 Finnish Institute for Molecular Medicine (FIMM), Helsinki University, Finland

**Keywords:** sequencing errors, 1000 Genomes data set, Illumina, next-generation sequencing, exome sequencing

## Abstract

It is often unavoidable to combine data from different sequencing centers or sequencing platforms when compiling data sets with a large number of individuals. However, the different data are likely to contain specific systematic errors that will appear as SNPs. Here, we devise a method to detect systematic errors in combined data sets. To measure quality differences between individual genomes, we study pairs of variants that reside on different chromosomes and co-occur in individuals. The abundance of these pairs of variants in different genomes is then used to detect systematic errors due to batch effects. Applying our method to the 1000 Genomes data set, we find that coding regions are enriched for errors, where ∼1% of the higher frequency variants are predicted to be erroneous, whereas errors outside of coding regions are much rarer (<0.001%). As expected, predicted errors are found less often than other variants in a data set that was generated with a different sequencing technology, indicating that many of the candidates are indeed errors. However, predicted 1000 Genomes errors are also found in other large data sets; our observation is thus not specific to the 1000 Genomes data set. Our results show that batch effects can be turned into a virtue by using the resulting variation in large scale data sets to detect systematic errors.

## Introduction

Next generation sequencing technologies allowed for the generation of data sets that include genetic data of a large number of individuals. To produce these data sets, sequencing data of different coverage, and from different platforms or different batches of sequencing chemistry may need to be combined. This can result in differences in the type and number of errors across samples (Wall et al. 2014; Wolpin et al. 2014; Schirmer et al. 2015; Torkamaneh et al. 2016; Kircher et al. 2011).

Here, we introduce a method to identify individual genomes with a higher error rate in large data sets and to predict which variants are likely due to error. The method first tests pairs of variants that reside on different chromosomes for signals of linkage disequilibrium. Linkage between separate chromosomes is not expected by population genetics theory for a randomly mating population, unless strong epistatic interactions are present. However, such signal can occur if errors affect individual genomes differently, leading to co-occurring erroneous variants in the same individuals but on different chromosomes (fig. 1). This first step is computationally expensive and we therefore limited the computation of linkage to pairs of variants in a subset of the genome. In the second step, we compare the contribution of individual genomes to the total linkage signal to identify outlier individuals that carry more potentially erroneous variants. As a last step, we use the differences in the number of linked pairs between individuals to identify which variants are present primarily in those individuals that carry more predicted errors (fig. 1). This last step can be applied to all variants and not only those that have been tested for linkage, resulting in a list of predicted erroneous variants for the complete data set. Removing these errors, we repeat the procedure starting from the second step, until no significant differences in the burden of predicted errors is observed between individuals. No knowledge of differences in sequencing technologies or other factors is required by this approach.

**FFig. 1.**
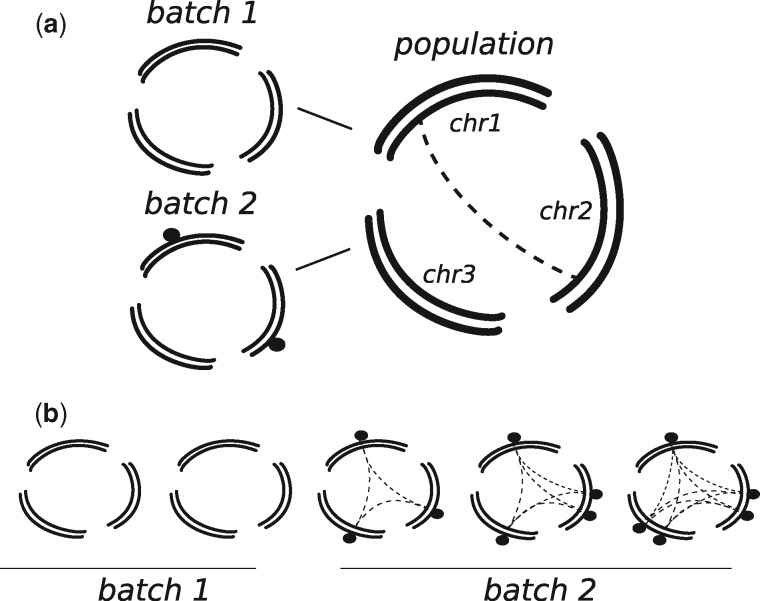
—Outline of the method. (*a*) Sequencing data generated from samples with different sequencing quality or processing might introduce different errors (black dots). Since these errors will be present in samples coming from the same platform, they will give a signal of linkage between different chromosomes (dashed lines). (*b*) The contribution to the linkage signal can be computed for each sample (dashed lines), and used to identify samples coming from the same batch and with similar error profiles, as well as the errors. See also supplementary figure 1, Supplementary Material online.

## Materials and Methods

### Data Handling

We downloaded the 1000 genomes phase 3 data set (version June 25, 2014). We used only one representative individual for each set of related individuals, using the 1000 genomes annotation. Only populations with at least 95 unrelated individuals were analyzed further, retaining 12 populations and 1,117 individuals (supplementary table 1, Supplementary Material online). Variants were classified according to frequency using bcftools (common variants: >5% frequency in at least one population, rare variants: 1% < frequency in at least one population, but ≤ 5% in all) (Li 2011). We performed all analyses on both common and rare variants, or only on common. Variants were annotated as coding when they fell within 200 bp of the coding exons of the UCSC known gene annotation (Rosenbloom et al. 2015), and as intergenic when they did not overlap UCSC known genes and were not annotated as a potentially functional variant by the Variance Effective Predictor (McLaren et al. 2016). The Botnia data include 327 trios from the Botnia population, in Finland (Fuchsberger et al. 2016). We excluded all offspring and related individuals.

Data from the Genome of the Netherlands were filtered and annotated analogously to the 1000 genomes. All analyses shown refer to variants with a 5% MAF cutoff.

### Outline of Pipeline

We implemented our analyses in a pipeline to detect interchromosomal linkage disequilibrium and detect variants affected by batch effects or inhomogeneity in the treatment of samples. This pipeline is outlined in supplementary figure 1, Supplementary Material online, and the different steps are described in the following sections.

#### Step 1: Linkage Disequilibrium

When the phase is unknown, as for two physically unlinked loci A and B with possible alleles A-a and B-b, respectively, a composite genotypic linkage disequilibrium can be calculated, by relying on a maximum likelihood estimate of the amount of AB-gametes that are present in samples. Following Weir (Weir 1996), we can arrange the counts of the nine possible observed genotypes for the two loci in a matrix:

**Table:** 

	B/B	B/b	b/b
A/A	n1	n2	n3
A/a	n4	n5	n6
a/a	n7	n8	n9

so that

(1) $$\Sigma_{AB}=2n_{1}+n_{2}+n_{4}+n_{5}/2.$$

The composite genetic disequilibrium equals *D*_AB_*= Σ*_AB_*/n*-2*p*_A_*p*_B_ where *n* is the number of samples. The sign of the composite linkage disequilibrium *D*_AB_ indicates whether alleles A and B (or a and b) occur preferentially in combination (*D*_AB_>0) or whether the alleles occurring most often together are A and b (or a and B) (*D*_AB_<0). We can test statistical association between two variants by either considering a two-tailed test (i.e., Fisher’s exact test, adopting normalization proposed by Kulinskaya and Lewin 2009), or by performing a 1-tailed Fisher’s exact test for the positive and negative associations between minor alleles, thus denoted as A and B.

In order to speed up calculations approximate *P* values were first determined with the χ^2^ based T2 method (Schaid 2004; Wu et al. 2008; Zaykin et al. 2008), and exact *P* values were calculated only for those pairs with an approximate *P* value < 100/*n*_SNP_^2^, where *n*_SNP_ is the total number of variants examined. While negative association between minor variants might also occur because of synergistic interaction between deleterious variants (Sohail et al. 2017), batch effects are expected to result in the positive association between errors introduced at low frequency (fig. 1). Thus, we restricted our analyses to significantly linked variants with a positive association. Note however, that batch effects can also result in an excess of negatively associated minor variants when high-frequency errors are present, as we observed in the coding regions of the 1000 Genomes data set (see supplementary fig. 21, Supplementary Material online, for an analysis of negative linkage, and supplementary fig. 22, Supplementary Material online, for both positive and negative linkage together).

With the exception of the per-population analyses shown in figure 2*a* and *b* and supplementary figures 6*a* and *b* and 10*c*, Supplementary Material online, we combine *P* values across populations, using Fisher’s method to obtain a single combined 1-tailed *P* value for each pair of variants. These combined *P* values are then compared with those obtained in a data set generated by randomly redistributing chromosomes across individuals. This allows one to additionally control for the sporadic linkage between chromosomes that can occur for low frequency alleles (Skelly et al. 2016). The False Discovery Rate was calculated as the fraction of allele-pairs that have an equal or lower *P* value in the randomized data set, versus the original data. In order to test the excess of interchromosomal linkage disequilibrium we restrict further analyses to instances in which at least one pair of variants is significantly associated (supplementary figs. 2 and 3, Supplementary Material online).

**FFig. 2.**
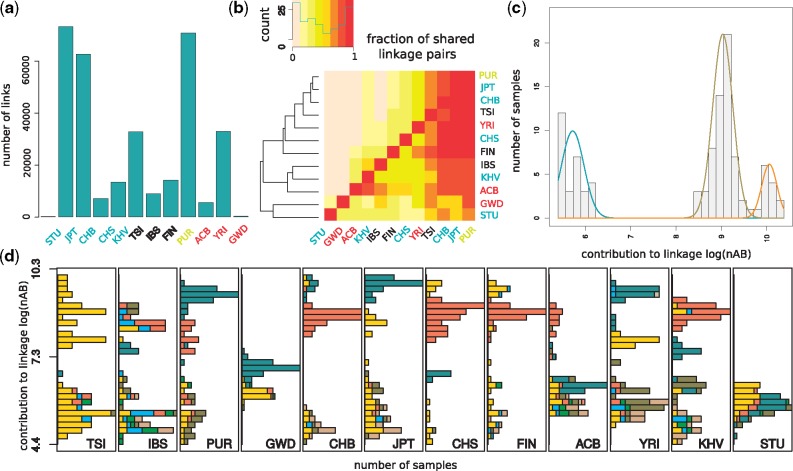
—Characteristics of interchromosomal linkage among common variants in the coding regions (>5% minor allele frequency). (*a*) Number of interchromosomal linked variants with a false discovery rate (FDR) < 5% in the 1000 Genomes populations, when analyzed independently. The FDR was calculated by comparing the *P* value of each linked pair to the distribution of *P* values after permuting chromosomes across individuals. Populations labels are colored according to the continent: blue for Asia, red for Africa, black for Europe and yellow for others. (*b*) Fraction of interchromosomal linked variants in one population (row) that are also linked in another population (column). Darker colors indicate a higher proportion of linked variants. The order of the populations is determined by the hierarchical clustering graph shown on the left, calculated on the basis of the sharing of linked variants. (*c*) Contribution of Chinese from Bejing individuals to the linkage signal (bars) given by the number of linked minor alleles (nAB). Individuals with similar nAB values were grouped by a Gaussian mixture model, whose fitted distributions are shown as colored lines. (*d*) Distribution of nAB for individuals from different 1000 Genomes populations. Colors indicate the sequencing center per individual. Individuals sequenced in multiple centers were marked with a separate colors.

#### Step 2: Individual Contribution to LD

We calculated the contribution of each sample/diploid individual to the linkage signal by summing up its contribution to the total of the $\Sigma_{AB}$ values over all significant linkage pairs. Note that this value, called nAB, is calculated per individual. For positive associations (*D* > 0), the contribution of each sample is directly the weight in equation (1) corresponding to a specific genotype configuration, for example, 2 if a sample has genotype n1 (A/A, B/B) since both gametes necessarily had alleles A and B on both chromosomes, and 0 if it has genotype n3 (A/A, b/b), since no gametes had alleles A and B on both chromosomes.

In order to test whether samples contribute uniformly to the linkage signal or not we compared the variance of the observed data and random reshuffling of the chromosomes across samples and within populations. To identify samples with similar features, we perform a finite mixture analysis (R package Mixtools), by assuming a normal distribution for the underlying model of the contribution to the linkage signal of each group of samples, within a population. We calculated the corrected Akaike Information Criterion (AICc) weights for each model, with one to nine possible underlying Gaussians for each population.

The models with the highest weights are shown in supplementary figure 7, Supplementary Material online. Since a Gaussian might deviate from the real underlying distribution, we tested whether a finite mixture analysis on the null data sets in which chromosomes are redistributed across individuals would provide less support to the presence of groups of samples with different nAB. We first calculated the relative support for the best supported model against the null model with only one Gaussian to explain the data, and compared it with the same statistics for 100 null data sets. Higher support for multiple clusters was present in the observed data compared with the null distribution (Wilcoxon-rank test, *P* value < 10^−16^ for coding regions, *P* value = 0.00103 for intergenic). Note that although generating permutations of the data is computationally expensive, the high number of potential links give a very narrow distribution of all statistics related to this empirical null distribution. For the 1000 genomes data set 3 randomizations are sufficient to provide highly significant *P* values when adopting a one-tailed *t*-test and comparing to the real data. When a data set showed a significantly higher variance and higher clustering than its empirical null distribution in terms of nAB, we proceed into identifying the variants responsible for the signal and the sources of the bias. In order to assess whether the identified clusters correspond to specific features of the samples we tested the role of several technical predictors extracted from the sample spreadsheet of the 1000 genomes data set (ftp://ftp.1000genomes.ebi.ac.uk/vol1/ftp/technical/working/20130606_sample_info/20130606_sample_info.xlsx). The clusters identified with Mixtools for the coding regions of the 1000 genomes are significantly associated with Sequencing Center and average coverage (combined χ^2^*P* value < 10^−16^) (supplementary figs. 8 and 9, Supplementary Material online).

#### Step 3: Identification of Error Candidates

In order to identify variants whose presence is explained by the occurrence in specific clusters of samples, we used a Generalized Linear Mixed Model iteratively on each variant, considering the contribution of each sample to nAB as only predictor. The underlying assumption is that samples that show a consistent excess of linked variants are more likely affected by technical artifacts. Hence, variants that are present only in these samples would be more likely spurious. To assess whether the presence of an allele is predicted by the contribution of each individual to the linkage signal, we built two models: a full model, including the nAB value for each individual as a predictor, and a null model, in which nAB is not included. We then compared the two models with a likelihood ratio test, so that for each variant we assess the significance of the relationship between nAB and the presence of the minor variant (supplementary fig. 11, Supplementary Material online). Notice, that this method uses as only predictor the observed linkage, and thus does not require any additional information about the samples. In more detail, the response variable of the linear models is the presence or absence of the minor allele per sample. A sample can have three states for this minor allele (absent homozygous a/a, heterozygous a/A or present homozygous A/A). To model nonindependence of the two chromosomes of each individual we consider each allele separately and introduce a predictive variable (factor) “Sample” that groups the two alleles for each chromosome of each individual (supplementary fig. 11, Supplementary Material online). The other predictor that is present in both the full and the null model is the population to which each individual belongs. In the full model, the contribution to the linkage signal per sample (nAB), is also present as a continuous covariate.

We can thus write the two models as:

- full model: presence allele A at site i ∼ contribution nAB + (1| Population) + (0 + contribution nAB | Population) + (1|Sample).
- null model: presence allele A at site i ∼ (1| Population) + (1|Sample).

Sample and Population were introduced as two random factors (categorical random predictors), in order to control for the nonindependence between chromosomes belonging to the same individual and individuals belonging to the same population. The effects of random factors are denoted as (1|Factor). The effects of a covariate, when dependent on a random factor, are denoted as (0 + covariate | Factor). In particular, we allowed for different effects of nAB in different populations (0 + contribution nAB | Population), due to potential differences in population composition and treatment.

The contribution of each sample to the nAB signal has been *z*-transformed and the *P* values of the likelihood ratio test are corrected for multiple testing with the Benjamini–Hochberg criterion. In order to speed up calculations we preliminarily scanned each sample with an analogous simpler logistic model, in which random factors are neglected, and populations are considered independently. The *P* values of each population are combined with Fisher’s method, and the full model including all random components was performed only for variants for which the combined *P* value was <0.01.

### Analysis of the 1000 Genomes Data Set

#### Characterization of Batch-Effects

To directly assess the association between nAB and technical features of the samples, we applied a Generalized Linear Mixed Model using the R package lme4 (supplementary tables 2 and 3, Supplementary Material online). We tested a model exploring whether the observed log(nAB) value for each sample (response variable) is predicted by technical features of the individual samples (predictor variables). As predictor variables, we used technical features of the samples described in the sample spreadsheet of the 1000 Genomes project. For simplicity, we grouped these different predictors into three main groups: Center, Coverage, and Chip. As a first predictor variable, we considered the main sequencing center where each sample was processed, that is, for coding regions the main sequencing center for the exome, and for intergenic regions the main sequencing center for the low coverage sequencing. For most samples, one sequencing center was used to produce all or at least the majority of the data, which was regarded as the main sequencing center; for the remaining cases (n < 3 for all populations), where equal proportions of data were produced at multiple sequencing centers, we considered this combination as an independent level. Center is a single categorical variable, in which the different levels of the linear model indicate different sequencing centers, and the coefficients estimated by the linear model (supplementary tables 2 and 3, Supplementary Material online) are the effect that each sequencing center has in respect to a baseline sequencing center selected from the spreadsheet. The second group of variables, Chip, includes three independent binary variables, each denoting whether one the genoyping array platforms (Omni, Affymetrix, or Axiom) was used for the sample. Finally, the group Coverage, describes the average coverage per sample, measured as three continuous variables from the sampled spreadsheet of the 1000 genomes data set, that is, Total.Exome.Sequence, X.Targets.Covered.to.20x.or.greater and LC.Non.Duplicated.Aligned.Coverage. Populations were included as random categorical predictors, and for all other predictor variables we considered random intercepts and random slopes nested within Population. This approach accounts for the different effects that the different predictor variables might have in different populations. We tested a full model, that included all predictor variables, and three reduced models including only some of the predictors: 1) the three continuous coverage variables (Coverage) + Center, 2) Coverage + the presence of genotyping arrays (Chip), 3) Center + Chip. The models were compared with a likelihood ratio test, indicating whether the group removed in the reduced model improves significantly the predictions of the model (supplementary tables 2 and 3, Supplementary Material online).

#### Idenfication of Error Candidates

We applied our method to all variants present in the 1000 genomes data sets. For the coding regions, where linkage pairs are abundant, we directly use the nAB values estimated exclusively on significantly linked variants using a minimum allele frequency threshold of 5% (supplementary table 4, Supplementary Material online) and 1% (supplementary table 5, Supplementary Material online). This procedure is underpowered for intergenic variants, where the amount of linked pairs is much smaller and the distribution of nAB has a low resolution. To increase the amount of bona fide linked variants in the intergenic data set, we first increased by ten-fold the number of pairwise interchromosomal comparisons by subsampling a larger amount of intergenic variants. In addition, we relax the FDR cutoff to define linked pairs to FDR < 20%. Notice that while this reduced cutoff may increase the noise in the nAB profile due to additional randomly linked pairs, we expect no systematic bias that would increase the number of predicted errors. In contrast, increasing the FDR cutoff for links considered to compute nAB has only a minimal effect on the number of predicted errors in coding regions, suggesting that the estimation of the nAB profile for the exome is not underpowered. The set of genome-wide discovered variants using linkage between intergenic variants is reported in supplementary tables 6 and 7, Supplementary Material online, for minor allele frequency cutoff of 5% and 1%, respectively. Note that supplementary tables 6 and 7, Supplementary Material online, include variants discovered genome-wide, also in regions for which the linkage was not computed directly.

For both data sets, we estimated the false discovery rate for each variant with the Benjamini–Hochberg method (supplementary tables 4–7, Supplementary Material online). An empirical false discovery measure can be obtained by calculating the overlap of the candidate variants from the 1000 genomes data set to variants present in Complete Genomics (supplementary figs. 14 and 15, Supplementary Material online). Significant variants for both data sets show a reduced overlap with the high quality Complete Genomics data set (*P* value < 10^−16^), indicating an enrichment in error among our candidates.

### Effects of Selection

In order to illustrate the possible selection scenarios that could lead to interchromosomal linked variants we calculated the dynamics in time of the average linkage-disequilibrium coefficient *D*, in presence of epistatic interactions between two different genomic variants leading to a difference in survival rates of the different gametes. We consider two biallelic sites, with alleles A-a, and B-b, respectively. We denote the number and selection coefficient of gametes AB, Ab, Ab, and aB with *n*_AB_, *n*_Ab_, *n*_aB_, and *n*_ab_, and *s*_AB_, *s*_Ab_, *s*_aB_, and *s*_ab_, respectively. We performed for each selection scenario 10,000 simulations. In each generation, we first simulated recombination, then selection.

In the recombination step, we sampled the number of gametes that would change state (i.e., gametes AB recombining with ab, and Ab recombining with aB) after random pairing of the gametes and recombination occurring with probability r. The selection step follows a Wright–Fisher model, with each gamete having fitness 1+*s*_AB_, 1+*s*_Ab_, 1+*s*_aB_, and 1+*s*_ab_, respectively. We consider two possible scenarios: advantageous combinations of minor alleles, with *s*_AB_>0 and *s*_Ab_=*s*_Ab_= *s*_ab_ =0, and antagonistic combinations of minor and major alleles (with *s*_AB_=*s*_ab_=0 and *s*_Ab_=*s*_aB_<0). Selection coefficients were either fixed to 1% (strong selection) or sampled from a distribution of selection coefficients estimated for nonsynonymous variants in the human genome (Racimo and Schraiber 2014). Simulations are shown in supplementary figure 5, Supplementary Material online.

### Validation of the Methods

We tested the current pipeline on simulated data sets with either 50, 100, or 200 unrelated individual genomes of equal length to that of the coding regions analyzed for the 1000 Genomes data set. The genotypes of these data sets were randomly sampled from the 1000 Genomes data sets. Each chromosome was sampled independently from the others, to obtain data sets with no residual linkage due to population structure nor errors. The individuals were divided into two batches, one with errors and one without errors. The error-containing batch encompassed either 20% or 50% of individuals. Errors were added to either 10% or 50% of the individuals of the error-containing batch at 0.1% of the sites. Errors were added in the form of false heterozygotes, leading to overall error rates equal between 10^−5^ and 0.000125 per site and individual. Results are shown in supplementary figures 24 and 25, Supplementary Material online. 

## Results

### Excess of Interchromosomal Linkage-Disequilibrium in the 1000 Genomes Data Set

We applied our method to the widely used 1000 Genomes data set (Sudmant et al. 2015; The 1000 Genomes Project Consortium 2015). The data used for the 1000 Genomes project have been acquired over 7 years, involving ten sequencing centers, five sequencing technologies, and several platform versions (The 1000 Genomes Project Consortium 2012, 2015). Individuals also differ in genome-wide sequencing coverage and in the coverage of the additional exome sequencing data. We limited our analysis to populations with at least 95 unrelated individuals, resulting in a total of 12 populations that we were able to test. Since many individuals from the 12 populations contained data generated via exome capture, we considered for our analysis all rare (minor allele frequency MAF >1% and <5%) and common variants (MAF > 5%) in coding regions (“coding region data set”; 107,087 sites over all 12 populations) and, as a separate data set (“intergenic data set”), an equal number of rare and common intergenic variants. To minimize the influence of population substructure on our analyses, we calculate intercchromosomal linkage for each population independently.

For both the intergenic and coding region data sets, and for all populations, we observe an excess of linked pairs over the expected number at a false discovery rate of 5%, or when comparing to an expectation derived from randomly assigning chromosomes to individuals (fig. 2*a*;supplementary figs. 2 and 3, Supplementary Material online). Analyzing each population separately, we find that linked pairs are often shared between populations, but this sharing does not reflect population relationships (fig. 2*b*). However, many more significant links are discovered in the coding region data set compared with the intergenic data set. In coding regions we find that variants are often linked to other variants on several different chromosomes, leading to large clusters of paired-variants (supplementary fig. 4, Supplementary Material online). Maintaining such large clusters would require implausible selective pressures that favor the coinheritance of minor alleles (supplementary fig. 5, Supplementary Material online). This contrasts with the concept of synergistic epistatic interaction among deleterious variants, which would lead to a repulsion between rare variants (Sohail et al. 2017).

Next, we calculated the contribution of each individual to the overall signal of linkage in a population by summing over the estimated number of linked pairs of minor alleles this individual carries (called nAB) (Weir 1996; Schaid 2004). For this, we considered all linked pairs that showed a significant combined *P* value across all populations (see Materials and Methods). We then compared the distribution of nAB over the individuals in a given population to the distribution calculated after randomly assigning chromosomes to individuals. We found that the variance in nAB is >80-fold higher in intergenic regions and >100-fold higher in coding regions compared with the expectation from randomization (Wilcoxon-rank test: intergenic *P* value < 7.4×10^−7^, coding region *P* value < 10^−20^), showing that the signal of linkage is driven by individuals that carry an excess of linked pairs. Interestingly, in coding regions most populations show groups of individuals that have similar nAB values, but differ from the values observed for individuals of other groups (fig. 2*c* and *d*;supplementary figs. 6 and 7, Supplementary Material online). Consistent with this observation, the nAB distribution in almost all populations fit a model of a mixture of several Gaussian distributions significantly better than a model with just one Gaussian distribution. We use the fitted Gaussians to assign individuals to groups (fig. 2*c*;supplementary figs. 6 and 7, Supplementary Material online).

### Identification of Errors

To explain the differences in nAB between individuals for the coding region data set, we correlated the group assignment of individuals from all populations with technical features of individuals annotated as part of the 1000 Genomes data set. We found that coverage, the presence of SNP array data for the sample and sequencing center are significantly associated with differences in nAB (coverage: *P* value < 10^−18^; sequencing center: *P* value < 10^−20^). Sequencing center has the strongest effect, explaining over 80% of the variation in nAB (fig. 2*d*;supplementary table 2 and figs. 8 and 9, Supplementary Material online): models including the predictor sequencing center explain up to 96% of the variation observed in the data, compared with a null model including only the predictor population that explains only ∼15% of the variation. Note however that different sequencing centers are often characterized by different average coverage per sample; besides, samples processed in certain sequencing centers also have additional genotyping array data. Thus, the predictors sequencing center, coverage, and SNP array are not independent (see also supplementary figs. 8 and 9, Supplementary Material online). For this reason, models including both the predictors coverage and SNP array but not the predictor sequencing center explain ∼79% of the observed variation in nAB. These results suggest that while known technical factors like coverage can explain part of the observed batch-effects (∼59% for exome sequencing coverage), additional technical processes affecting nAB are captured here by the predictor sequencing center, and account for ∼20% of the variation in nAB. We notice that alternative explanations are incompatible with the observed signal: for example, the possibility that polymorphic genetic rearrangements contribute to the linkage signal to a large extent is incompatible with the clustering of individuals according to their nAB, whereas population substructure would not generate the same linked variants across different populations. For the intergenic data set, we find that sequencing center is still strongly associated with nAB, but coverage is only marginally associated when considering a minor allele frequency of 1% (supplementary table 3, Supplementary Material online). Consistently, models including sequencing center are better supported than models that do not include it but only include the predictors coverage and SNP array (*P* value < 10^−^^16^), and explained a larger proportion of the variation in nAB (42.2–44.1% and 25.8% for models including or not the predictor sequencing center, respectively, for variants with allele frequency >5%). Much fewer variants appear linked in the intergenic compared with the coding region data set (421 or ∼1% of the number of linked pairs in the coding regions; see fig. 2*a* vs supplementary fig. 10*c*, Supplementary Material online) across all populations. A larger amount of intergenic variants to determine linked pairs does not change this result.

We next searched for variants where a minor allele is preferentially encountered in individuals with a high nAB value (supplementary fig. 11, Supplementary Material online). Genome-wide (coding regions and noncoding regions) we identify 16,951 common variants (>5% MAF in at least one population) in the 1000 Genomes data that are significantly associated with nAB and form our set of error candidates. Interestingly, these candidates are not distributed randomly over the genome, but are enriched in coding regions, where around 696 variants (∼1%) are predicted to be errors (supplementary tables 4 and 5, Supplementary Material online). In comparison, in noncoding regions only a small fraction (<0.001%) were labeled as candidates, even if more variants are sampled to increase power in the prediction (supplementary tables 6 and 7, Supplementary Material online). To further test the enrichment in coding regions, we used the software *admixture* (Alexander et al. 2009), which labels individuals by components of ancestry, on variants in coding regions and on all variants genome-wide. Coding regions showed components that corresponded to the grouping of individuals by nAB and with technical features of the samples (supplementary fig. 12, Supplementary Material online), while such an effect was not observed for noncoding regions variants, suggesting that variants in coding regions are enriched for error.

### Presence of Error Candidates in Different Data Sets

We would expect that our predicted errors are shared less often than real variants with data sets produced at high coverage by different technologies. To test this prediction, we calculate how often our candidate variants are found in the genomes of 69 individuals generated by Complete Genomics and compare this number to the sharing of other frequency matched variants from the 1000 Genomes data set. In coding regions, 85% of the matched variants are found in the Complete Genomics data sets, while only 15% of our candidates are shared (χ^2^ test *P* value < 10^−15^). In noncoding regions 80% of variants match, while only 56% of candidates are shared (*P* value < 10^−15^). This suggests that ∼84% of our predicted variants in coding regions and 33% of variants in noncoding regions are more likely due to error, assuming conservatively that Complete Genomics is devoid of errors that are shared with the 1000 Genomes data set. These proportions increase for lower FDR thresholds and lower allele frequencies (supplementary figs. 14 and 15, Supplementary Material online). For example, only 42 out of 4,681 candidates in noncoding regions that have a frequency <1% are present in Complete Genomics, and 98% of these candidates are estimated to be errors; the fraction of true errors is 56% when considering all variants <10% frequency.

We also assessed whether these errors are unique to the 1000 Genomes data set or whether they can be found in other large collections of genomes that may contain similar batch effects. We find that 7,843 error candidates, of which 69 occur in coding regions, are also present in the HRC data set (The Haplotype Reference Consortium 2016) In the GoNL data set (The Genome of the Netherlands Consortium et al. 2014) we find 7,380 error candidates, of which 32 are in coding regions. These variants are underrepresented in the Complete Genomics data set (χ^2^-test <10^−16^ for both GoNL and HRC), although with a proportion of estimated errors lower than those of the full set of candidates (49% and 17% in the coding and noncoding regions in GoNL; 69% and 26% in HRC). We also note that while we are able to detect some of the systematic errors from the 1000 Genomes in both data sets, the fraction of predicted errors that are present (56% and 0.49% in GoNL and HRC, respectively) is significantly lower than the fraction of variants that were not labeled as error (67% and 80%; *P* values < 10^−15^ in both cases). For the HRC data set, which is based on 1000 Genomes data, this suggests that further filtering was efficient in removing a large proportion of systematic errors. Consistenly, in the coding regions only 10% of the candidates are present in both 1000 Genomes and HRC data sets, while 91% of the frequency matched coding variants overlap.

### Characterizing the Features of Predicted Errors

Errors may be caused by a variety of technical issues (Dohm et al. 2008; Schirmer et al. 2015; Chen et al. 2017; Wang et al. 2017). To learn more about the features of error candidates in the 1000 Genomes data set, we tested several characteristics in comparison to background variants that were randomly selected from the set of all tested variants. We first tested candidates in coding region, and divided the candidates and control variants into synonymous and nonsynonymous sites. Whereas the control set shows an approximatively equal number of nonsynonymous and synonymous variants (∼48% nonsynonymous), error candidates show a much higher proportion of nonsynonymous variants (∼72%), consistent with the expected fraction of nonsynonymous substitutions that would be generated by random errors, given the codon composition of human genes (∼72%) (Nei and Gojobori 1986) (fig. 3). Coding region candidates also show a 2-fold higher proportion of transversions (*z*-test *P* < 10^−6^), a base composition with a 75% higher proportion of Gs and Cs (*z*-test *P* < 10^−12^, 70% GC content for error candidates vs 40% for background SNPs), a 4.2-fold higher propensity to fall within short C or G homopolymer stretches (*z*-test *P* < 10^−6^), and an ∼125-fold higher proportion of SNPs showing an excess of heterozygous genotypes in respect to the Hardy–Weinberg equilibrium (Wilcoxon-rank test on control and candidate errors; *P* values: *P* < 10^−12^), compared with background SNPs (fig. 3). The last test indicates that erroneous sites are often heterozygous. Allele imbalance, that is, the unequal representation of sequences supporting the two alleles in a high-coverage sample, is often used as a hallmark sign for erroneous heterozygotes (Li 2014). To test whether this is also true for our candidates we used 25 high-coverage samples from the Simons Genome Diversity Panel (SGDP) panel (Mallick et al. 2016), which were independently sequenced with the Illumina platform. Specifically, we tested allele imbalance in potential heterozygous samples of the SGDP panels, that is, samples showing at least one read in support of the reference allele and at at least one read in support of the alternative allele, at positions classified as error candidates in the 1000 Genomes Project data set or for background SNPs. While 81.4% of the error candidates showed a significant (*P* value < 0.05) deviation from the expected 50% proportion of reads supporting the alternative and the reference allele (binomial test with probability = 0.5), only 9.1% of the background SNPs showed a significant deviation, indicating a strong enrichment in samples displaying allele imbalance (χ^2^ test *P* value <10^−12^). This suggests that sequencing errors or cross-contamination introduce apparent heterozygous sites (fig. 3 and supplementary fig. 16, Supplementary Material online). For genome-wide candidates, we find some of the same signals, albeit often weaker. Candidates still exhibit a 2-fold higher tendency to reside in homopolymer stretches (*z*-test *P* < 10^−5^), occur >8 times more often in heterozygous state (*P* < 10^−12^) and show allele imbalance (*P* < 10^−12^). Furthermore, both candidates in intergenic and coding regions (both with χ^2^*P* value < 10^−16^) overlap repeats more often than background candidates (fig. 4). As part of their release, the 1000 Genomes Project provided users with two annotations, that label variants by high or low coverage, or the presence of low mapping quality sequences. These two annotations differ in strictness, with one representing more permissive criteria that label fewer bases as potentially problematic (pilot accessibility filter) and the other a stricter filter that labels more variants (strict accessibility filter). We observe that the majority of error candidates in coding regions are not labeled by either annotation, whereas at least 25% remain unlabeled for genome-wide variants (fig. 4). This indicates that, at least in coding regions, interchromosomal linkage detects erroneous variants that are not detectable when considering coverage or mapping quality alone.

**FFig. 3.**
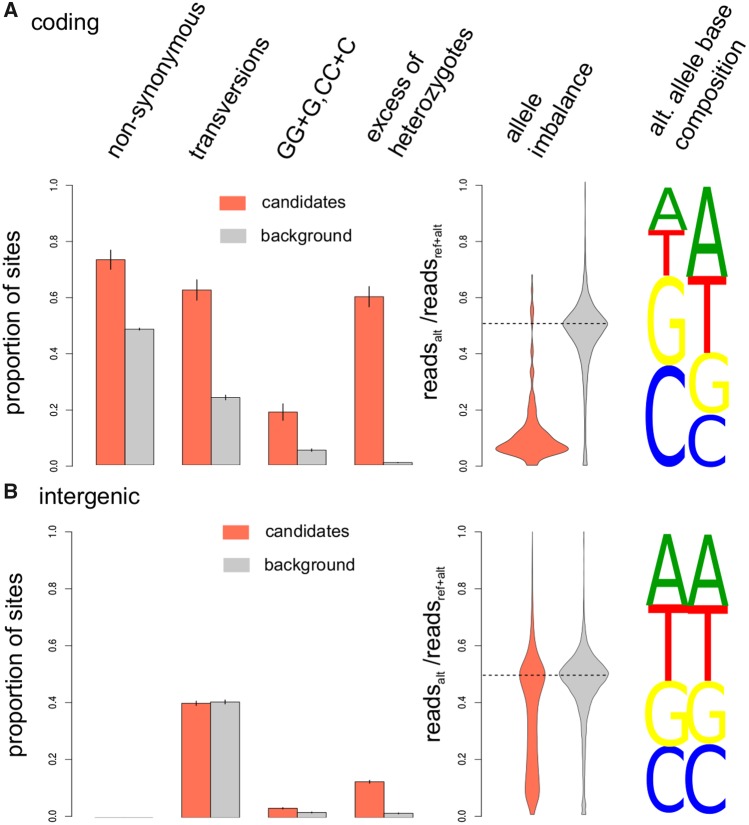
—Characteristics of error candidates in the 1000 Genomes data set detected in coding regions (*a*) or genome-wide based on the intergenic data set (*b*). For error candidates (red) and frequency-matched background variants (gray), the barplot shows the proportions of nonsynonymous versus synonymous variants, transversions versus transitions, alternative alleles introducing Gs or Cs after or before GG or CC dimers, and positions with significant excess of heterozygotes (*P* value < 0.05). The violin plot shows the proportion of sequences supporting the alternative alleles in individual with at least one sequence showing the alternative allele. On the right, the base composition of alternative alleles is shown for error candidates (red) and background variants (gray).

**FFig. 4.**
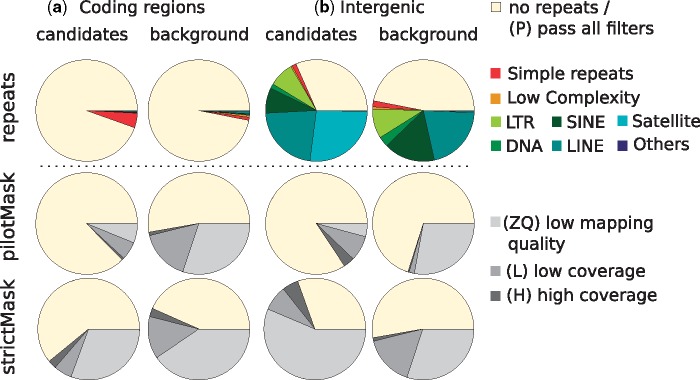
—Overlap of error candidates (left) and background variants (right) with repeated regions (top) in the genome and accessibility filters provided by the 1000 Genomes Projects (middle and bottom). Candidates and 20,000 background variants detected using the minor allele frequency filter ≥5% were overlapped with known repeats in RepeatMasker (http://www.repeatmasker.org; last accessed September 1, 2017) annotation for hg19 downloaded from UCSC genome browser, and two filters from the 1000 Genomes Project considering coverage and mapping quality. The coverage filters exclude regions where the depth of coverage (summed across all samples) was higher or lower than the average depth by a factor of 2-fold (pilotMask) or by 50% (strictMask). Regions are deemed as lowly mappable if >20% of overlapping reads have mapping quality of zero (pilotMask) or >0.1% (strictMask). LTR, RNA related repeats, and repeats classified as “unknown” or “others” by RepeatMasker are here labeled as Others, in dark blue.

Interestingly, we find that only the strict accessibility mask is enriched for candidate errors, compared with background variants when considering intergenic variants (χ^2^ test, *P* value < 10^−16^). No significant enrichment is observed for the pilot accessibility filter for intergenic variants and neither strict nor pilot accessibility filters show significant enrichment for coding region error candidates. Note that the pilot accessibility filter masks sites where >20% of sequences align with a mapping quality of 0. This criterion is likely ineffective for low frequency variants that constitute the majority of our predicted errors. Besides, both coverage accessibility filters provided by the 1000 Genomes Project are solely based on the low-coverage sequencing data, and do not include information about the exome data, that account on an average for ∼8 times more sequencing reads. Thus, errors in coding regions that are caused primarily by the exome sequencing data are not expected to be captured by these filters.

To further test whether error candidates in coding regions are primarily linked to exome capture and not shotgun sequencing data, we downloaded all sequences of the 1000 Genomes Project for one population (CHB). We then compared the proportion of sequences supporting the minor allele at positions classified as candidate errors and at 5,000 background variable positions in the coding regions, in low-coverage and exome sequencing data. We observed a 25.7% higher proportion of sequences supporting the minor allele in the exome data compared with the low-coverage data, while there is no difference for background variants (binomial test, *P* value = 0.978), supporting an enrichment in sites with different error rates in exome and low-coverage sequencing among our candidates (χ^2^ test, *P* value = 0.32×10^−7^). Note that different individuals have different fractions of sequencing reads produced via exome sequencing compared with the total of all sequences covering the exome. These fractions range per individual from a minimum of 46.8% to a maximum of 99.9%. This means that for certain variants, many individual genotypes are based almost exclusively on exome sequencing data, while others have higher proportions of low-coverage data, potentially introducing batch-effects. Interestingly, for candidate errors the coverage of the exome across individuals has a 15.4% higher standard deviation than background variants. This is consistent with the findings that the coverage of the exome is a strong predictor of the contribution to the linkage signal (supplementary table 1 and fig. 8, Supplementary Material online).

### Testing Uniform Data Sets

Our method predicts errors that likely originate from the combination of technologies, but should not find errors when the data set is generated with just one technology and does not contain other batch effects. To test this prediction, we analyzed data from the GoNL data set (The Genome of the Netherlands Consortium et al. 2014), which is composed of two parts that were produced with the Illumina and Complete Genomics (Drmanac et al. 2010) sequencing technologies, respectively. We first analyzed the two parts independently and found no excess of linked pairs, consistent with a uniform error within each part. However, when both parts are merged, 2,750 linkage pairs with FDR < 20% are detected (supplementary fig. 17 and table 8, Supplementary Material online), representing batch-specific variants that are likely due to error. We applied our method to detect the variants that drive this signal. Similar to the 1000 genomes data set the identified variants display typical features of errors, such as an excess of heterozygotes and evidence of allele imbalance (supplementary fig. 18, Supplementary Material online).

We also analyzed how many false positive errors we predict in another uniform data set containing 654 unrelated individuals from the Botnia region (Fuchsberger et al. 2016). The data set was produced as part of a diabetes genome-wide association study using OmniChip. Our method found no excess of linked pairs in this data set and the distribution of nAB across individuals is comparable to that observed after randomly permuting chromosomes across individuals.

## Discussion

Previous studies used local patterns of linkage disequilibrium (LD) in order to improve the quality of haplotype and SNP calls in large-scale studies (Scheet and Stephens 2006; Leek 2014). An example is the fastPhase method, which allowed for the identification of over 1,500 low frequency SNPs with high error rates in the HapMap data sets (Scheet and Stephens 2006). Our method uses a different source of information and can be combined with these approaches to predict errors. Here, we have shown that long-distance linkage between pairs of sites that reside on different chromosomes can be used to predict individuals that show an excess of error and to label variants that are likely errors.

The errors we detected can influence a variety of analyses. For instance, we showed that they affect estimates of population structure (supplementary fig. 12, Supplementary Material online) and estimates of mutational load (supplementary figs. 19 and 20, Supplementary Material online). Furthermore, since exons are enriched for errors and random errors appear more often as nonsynonymous variants, estimates of functional mutational load and the fitness effects of newly arising mutations might be affected. The apparent linkage between chromosomes can also affect studies of epistatic interactions. For example, Sohail et al. were able to detect epistatic effects only for the most functional elements of the genomes, and detected an overall signal of linkage disequilibrium compatible with the presence of errors, as identified in the present study (Sohail et al. 2017). Our approach allows us to identify these errors.

We note that our estimates of the per-individual errors allow for further analyses to study the possible origin of batch effects. For example, we observed in the 1000 Genomes data set a strong effect of the sequencing center, followed by coverage and genotyping array used (supplementary tables 2 and 3 and figs. 8 and 9, Supplementary Material online). However, the insights from the published metainformation are limited since sequencing center, for instance, could represent a variety of underlying causes for quality differences, such as a differences in chemical reagents or operating conditions (Leek et al. 2010; Chen et al. 2017).

Our error candidates showed an excess of heterozygotes. These heterozygotes are characterized by allele imbalance in independently sequenced high-coverage data sets. This suggests that these positions are susceptible to recurrent errors. Note that these errors are elusive, and often not captured by coverage and mappability based filters. We note that our analysis was restricted to variants that passed genotype quality filters (labeled as PASS). However, consistently with the signal of allele imbalance, the genotype quality of these heterozygous errors was on an average a bit lower than for other variants (supplementary fig. 20, Supplementary Material online).

Concluding, interchomosomal linkage disequilibrium leverages the usage of different technologies to identify errors that could remain unidentified when only one technology is used for sequencing. We showed this using the GoNL data set, for which combining two data sets generated with different technologies allows for the discovery of platform-specific errors. Thus, while using a single platform may help in obtaining data sets with errors that are comparable between samples, the combination of these data sets can help identify errors that are different between technologies. We hope that our method helps to increase the value of large-scale heterogeneous data sets that are more susceptible to batch-effects.

## Supplementary Material

Supplementary Data

## References

[evy199-B1] Alexander DH, Novembre J, Lange K. 2009 Fast model-based estimation of ancestry in unrelated individuals. Genome Res. 19:1655–1664. 19648217 10.1101/gr.094052.109 PMC2752134

[evy199-B2] Chen L, Liu P, Evans TC, Ettwiller LM. 2017 DNA damage is a pervasive cause of sequencing errors, directly confounding variant identification. Science 355(6326):752–756. 28209900 10.1126/science.aai8690

[evy199-B3] Dohm JC, Lottaz C, Borodina T, Himmelbauer H. 2008 Substantial biases in ultra-short read data sets from high-throughput DNA sequencing. Nucleic Acids Res. 36(16):e105. 18660515 10.1093/nar/gkn425 PMC2532726

[evy199-B4] Drmanac R, et al 2010 Human genome sequencing using unchained base reads on self-assembling DNA nanoarrays. Science 327(5961):78–81. 19892942 10.1126/science.1181498

[evy199-B5] Fuchsberger C, et al 2016 The genetic architecture of type 2 diabetes. Nature 536(7614):41–47. 27398621 10.1038/nature18642 PMC5034897

[evy199-B6] Kircher M, Heyn P, Kelso J. 2011 Addressing challenges in the production and analysis of illumina sequencing data. BMC Genomics 12(1):382. 21801405 10.1186/1471-2164-12-382 PMC3163567

[evy199-B7] Kulinskaya E, Lewin A. 2009 Testing for linkage and Hardy-Weinberg disequilibrium. Ann Hum Genet. 73(2):253–262. 19183345 10.1111/j.1469-1809.2008.00501.x

[evy199-B8] Leek JT. 2014 svaseq: removing batch effects and other unwanted noise from sequencing data. Nucleic Acids Res. 42(21):e161. 10.1093/nar/gku864 PMC4245966 25294822

[evy199-B9] Leek JT, et al 2010 Tackling the widespread and critical impact of batch effects in high-throughput data. Nat Rev Genet. 11(10):733–739. 20838408 10.1038/nrg2825 PMC3880143

[evy199-B10] Li H. 2011 A statistical framework for SNP calling, mutation discovery, association mapping and population genetical parameter estimation from sequencing data. Bioinformatics 27(21):2987–2993. 21903627 10.1093/bioinformatics/btr509 PMC3198575

[evy199-B11] Li H. 2014 Toward better understanding of artifacts in variant calling from high-coverage samples. Bioinformatics 30(20):2843–2851. 24974202 10.1093/bioinformatics/btu356 PMC4271055

[evy199-B12] Mallick S, et al 2016 The Simons Genome Diversity Project: 300 genomes from 142 diverse populations. Nature 538(7624):201–206. 27654912 10.1038/nature18964 PMC5161557

[evy199-B13] McLaren W, et al 2016 The Ensembl variant effect predictor. Genome Biol. 17(1):122. 27268795 10.1186/s13059-016-0974-4 PMC4893825

[evy199-B14] Nei M, Gojobori T. 1986 Simple methods for estimating the numbers of synonymous and nonsynonymous nucleotide substitutions. Mol Biol Evol. 3:418–426. 3444411 10.1093/oxfordjournals.molbev.a040410

[evy199-B15] Racimo F, Schraiber JG. 2014 Approximation to the distribution of fitness effects across functional categories in human segregating polymorphisms. PLoS Genet. 10(11):e1004697. 25375159 10.1371/journal.pgen.1004697 PMC4222666

[evy199-B16] Rosenbloom KR, et al 2015 The UCSC Genome Browser database: 2015 update. Nucleic Acids Res. 43(D1):D670–D681. 25428374 10.1093/nar/gku1177 PMC4383971

[evy199-B17] Schaid DJ. 2004 Linkage disequilibrium testing when linkage phase is unknown. Genetics 166(1):505–512. 15020439 10.1534/genetics.166.1.505 PMC1470678

[evy199-B18] Scheet P, Stephens M. 2006 A fast and flexible statistical model for large-scale population genotype data: applications to inferring missing genotypes and haplotypic phase. Am J Hum Genet. 78(4):629–644. 16532393 10.1086/502802 PMC1424677

[evy199-B19] Schirmer M, et al 2015 Insight into biases and sequencing errors for amplicon sequencing with the Illumina MiSeq platform. Nucleic Acids Res. 43(6):e37. 25586220 10.1093/nar/gku1341 PMC4381044

[evy199-B20] Skelly DA, Magwene PM, Stone EA. 2016 Sporadic, global linkage disequilibrium between unlinked segregating sites. Genetics 202(2):427–437. 26715671 10.1534/genetics.115.177816 PMC4788226

[evy199-B21] Sohail M, et al 2017 Negative selection in humans and fruit flies involves synergistic epistasis. Science 356(6337):539–542. 28473589 10.1126/science.aah5238 PMC6200135

[evy199-B22] Sudmant PH, et al 2015 An integrated map of structural variation in 2,504 human genomes. Nature 526(7571):75–81. 26432246 10.1038/nature15394 PMC4617611

[evy199-B23] The 1000 Genomes Project Consortium. 2015 A global reference for human genetic variation. Nature 526:68–74. 26432245 10.1038/nature15393 PMC4750478

[evy199-B24] The 1000 Genomes Project Consortium. 2012 An integrated map of genetic variation from 1,092 human genomes. Nature 491(7422):56–65. 23128226 10.1038/nature11632 PMC3498066

[evy199-B25] The Genome of the Netherlands Consortium, et al 2014 Whole-genome sequence variation, population structure and demographic history of the Dutch population. Nat Genet. 46:ng.3021. 10.1038/ng.3021 24974849

[evy199-B26] The Haplotype Reference Consortium. 2016 A reference panel of 64,976 haplotypes for genotype imputation. Nat Genet. 48:1279–1283. 27548312 10.1038/ng.3643 PMC5388176

[evy199-B27] Torkamaneh D, Laroche J, Belzile F. 2016 Genome-wide SNP calling from genotyping by sequencing (GBS) data: a comparison of seven pipelines and two sequencing technologies. PLoS One 11(8):e0161333. 27547936 10.1371/journal.pone.0161333 PMC4993469

[evy199-B28] Wall JD, et al 2014 Estimating genotype error rates from high-coverage next-generation sequence data. Genome Res. 24(11):1734–1739. 25304867 10.1101/gr.168393.113 PMC4216915

[evy199-B29] Wang B, Wan L, Wang A, Li LM. 2017 An adaptive decorrelation method removes Illumina DNA base-calling errors caused by crosstalk between adjacent clusters. Sci Rep. 7(1):41348. 28216647 10.1038/srep41348 PMC5316982

[evy199-B30] Weir BS. 1996. Genetic data analysis II: methods for discrete population genetic data. Sunderland (MA): Sinauer Associates. p. 91–139.

[evy199-B31] Wolpin BM, et al 2014 Genome-wide association study identifies multiple susceptibility loci for pancreatic cancer. Nat Genet. 46(9):994–1000. 25086665 10.1038/ng.3052 PMC4191666

[evy199-B32] Wu X, Jin L, Xiong M. 2008 Composite measure of linkage disequilibrium for testing interaction between unlinked loci. Eur J Hum Genet. 16(5):644–651. 18212814 10.1038/sj.ejhg.5202004

[evy199-B33] Zaykin DV, Pudovkin A, Weir BS. 2008 Correlation-based inference for linkage disequilibrium with multiple alleles. Genetics 180(1):533–545. 18757931 10.1534/genetics.108.089409 PMC2535703

