# Supplementary material for: Turning Vice into Virtue: Using Batch-Effects to Detect Errors in Large Genomic Data Sets
Source: Genome Biol Evol. 2018 Sep 10;10(10):2697–708. doi: 10.1093/gbe/evy199 (PMC6185451; doi:10.1093/gbe/evy199)
Supplement: Supplementary Data [file evy199_supp.zip › SI.pdf]

Supplementary Tables and Figures

a)

Summary Full model with MAF >5%	Estimate	Std. Error	t value	Factor
(Intercept)	-1,494	0,175	-8,523	
Total.Exome.Sequence	0,055	0,054	1,032	Coverage
X..Targets.Covered.to.20x.or.greater	-0,113	0,050	-2,265	Coverage
LC.Non.Duplicated.Aligned.Coverage	-0,036	0,021	-1,706	Coverage
Main.Project.E.Center A+B	1,829	0,368	4,971	Center
Main.Project.E.Center B	1,629	0,159	10,263	Center
Main.Project.E.Center C	1,991	0,239	8,342	Center
Main.Project.E.Center D	0,656	0,155	4,227	Center
Has.Affy.6.0.Genotypes	-0,568	0,243	-2,334	Chip
Has.Axiom.Genotypes	0,403	0,184	2,188	Chip

Model	Df	AIC	BIC	loglik	R2	χ2	Df	Pr(>χ2)
Coverage+Center+Chip (Full model)	66	97,5	434,4	17,2	0,960			
Coverage+Center	45	489,5	719,2	-199,8	0,940	433,97	21	<10^-16
Coverage+Chip	28	2189,6	2332,5	-1066,8	0,791	2168,1	38	<10^-16
Center+Chip	36	357,3	541,1	-142,7	0,960	319,81	30	<10^-16
Null (only population)	13	3276	3342,3	-1625,0	0,152	3284,4	53	<10^-16

b)

Summary Full model with MAF >1%	Estimate	Std. Error	t value	Factor
(Intercept)	-1,568	0,217	-7,228	
Total.Exome.Sequence	0,057	0,049	1,157	Coverage
X..Targets.Covered.to.20x.or.greater	-0,151	0,051	-2,949	Coverage
LC.Non.Duplicated.Aligned.Coverage	-0,023	0,022	-1,055	Coverage
Main.Project.E.Center A+B	1,546	0,375	4,120	Center
Main.Project.E.Center B	1,633	0,137	11,913	Center
Main.Project.E.Center C	2,056	0,214	9,626	Center
Main.Project.E.Center D	0,674	0,156	4,333	Center
Has.Affy.6.0.Genotypes	-0,393	0,245	-1,604	Chip
Has.Axiom.Genotypes	0,447	0,226	1,982	Chip

Model	Df	AIC	BIC	loglik	R2	χ2	Df	Pr(>χ2)
Coverage+Center+Chip (Full model)	66	503,1	840,0	-185,6	0,946			
Coverage+Center	45	767,5	997,2	-338,8	0,923	306,37	21	<10^-16
Coverage+Chip	28	2263,7	2406,6	-1103,9	0,795	1836,6	38	<10^-16
Center+Chip	36	672,0	855,7	-300,0	0,911	228,82	30	<10^-16
Null (only population)	13	3283,9	3350,2	-1628,9	0,147	2886,7	53	<10^-16

Supplementary Table 2: Linear-Mixed-Model analyses on coding regions of the 1000 genomes dataset, for alleles with a minor frequency threshold of (a) 5% or (b) 1%. The coefficients (Estimate), errors (Std.Error) and t-values of the full model including the predictors Center, Chip, and Coverage (Factor) are reported in the top panels. The categorical predictor Center consists of different levels – the different sequencing centers in which a sample was processed – for which the effects in respect to a baseline sequencing center are estimated. Thus the effects of the baseline sequencing center are included in the intercept. The other coefficients represent the contribution to the log(nAB) of the presence of a specific genotyping array (Factor Chip) or caused by an increase in 1 standard deviation of any of the predictors labeled as Coverage in the column Factor.

To test explicitly the effects of the different variables, we tested the full model against reduced models in which one of the variable is removed (Model). Three different continuous variables are grouped together under the label Coverage, and excluded together when comparing the full model

to the reduced model (Center+Chip). Analogously, three different categorical variables are grouped together under the label Chip, and excluded together when comparing the full model to the reduced model (Coverage+Center). We also report the comparison of the full model with a null model in which only the predictor population is included. Akaike Information Criterion (AIC) values, Bayesian Information Criterion (BIC), log likelihood, the variance explained by the model (r^2 coefficient computed with the package R MuMIn) and p-value according to a likelihood ratio test comparing full and reduced model are shown below. The first Df column indicates the degrees of freedom of the model, the second the difference in degrees of freedom. Information relative to the different variables can be found in the sample spreadsheet of the 1000 Genomes dataset (ftp://ftp.1000genomes.ebi.ac.uk/vol1/ftp/technical/working/20130606_sample_info/20130606_sample_info.xlsx). For coding regions, the sequencing centers per sample considered are those reported in the Main.project.LC.Center field of the sample spreadsheet.

a)

Summary Full model with MAF >5%	Estimate	Std. Error	t value	Factor
(Intercept)	-0,631	0,124	-5,075	
Total.Exome.Sequence	0,067	0,030	2,222	Coverage
X..Targets.Covered.to.20x.or.greater	-0,111	0,037	-3,029	Coverage
LC.Non.Duplicated.Aligned.Coverage	0,085	0,051	1,663	Coverage
Main.project.LC.Center A+B	1,676	0,771	2,172	Center
Main.project.LC.Center B	1,467	0,156	9,399	Center
Main.project.LC.Center C	0,487	0,143	3,399	Center
Main.project.LC.Center D+F	2,455	0,764	3,214	Center
Main.project.LC.Center G	0,263	0,211	1,247	Center
Main.project.LC.Center F	0,247	0,174	1,421	Center
Main.project.LC.Center H	0,621	0,169	3,670	Center
Main.project.LC.Center D	-0,437	0,166	-2,630	Center
Has.Affy.6.0.Genotypes	0,064	0,090	0,705	Chip
Has.Axiom.Genotypes	-0,212	0,090	-2,349	Chip

Model	Df	AIC	BIC	loglik	R2	χ2	Df	Pr(>χ2)
Coverage+Center+Chip (Full model)	120	3029,3	3641,8	-1394,7	0,453			
Coverage+Center	91	2979,7	3444,2	-1398,9	0,441	8,4288	29	0,9999
Coverage+Chip	28	3151,9	3294,8	-1548,0	0,258	306,59	92	<10^-16
Center+Chip	78	2981,8	3380,0	-1412,9	0,422	36,515	42	0,7099
Null (only population)	13	3346,9	3413,2	-1660,4	0,102	531,57	107	<10^-16

b)

Summary Full model with MAF >1%	Estimate	Std. Error	t value	Factor
(Intercept)	-0,408	0,172	-2,380	
Total.Exome.Sequence	-0,007	0,039	-0,169	Coverage
X..Targets.Covered.to.20x.or.greater	0,007	0,060	0,118	Coverage
LC.Non.Duplicated.Aligned.Coverage	0,111	0,040	2,753	Coverage
Main.project.LC.Center A+B	0,141	0,743	0,190	Center
Main.project.LC.Center B	1,232	0,149	8,261	Center
Main.project.LC.Center C	0,300	0,126	2,380	Center
Main.project.LC.Center D+F	0,008	0,705	0,012	Center
Main.project.LC.Center G	0,016	0,206	0,079	Center
Main.project.LC.Center F	-0,041	0,175	-0,237	Center
Main.project.LC.Center H	0,451	0,106	4,246	Center
Main.project.LC.Center D	-0,281	0,138	-2,034	Center
Has.Affy.6.0.Genotypes	0,219	0,112	1,954	Chip
Has.Axiom.Genotypes	-0,119	0,162	-0,734	Chip

Model	Df	AIC	BIC	loglik	R2	χ2	Df	Pr(>χ2)
Coverage+Center+Chip (Full model)	120	2861,8	3474,3	-1310,9	0,488			
Coverage+Center	91	2820,1	3284,6	-1319,0	0,502	16,301	29	0,972
Coverage+Chip	28	2962,7	3105,6	-1453,3	0,466	284,9	92	<10^-16
Center+Chip	78	2833,7	3231,8	-1338,8	0,469	55,896	42	0,07405
Null (only population)	13	3067,6	3134,0	-1520,8	0,285	419,84	107	<10^-16

Supplementary Table 3: Linear-Mixed-Model analyses of intergenic regions of the 1000 Genomes Project dataset, for alleles with a minor frequency threshold of (a) 5% or (b) 1%. Description as in Supplementary Table 2. For intergenic regions, the sequencing centers considered for each sample are those reported in the Main.project.LC.Center field of the sample spreadsheet of the 1000 Genomes Project.

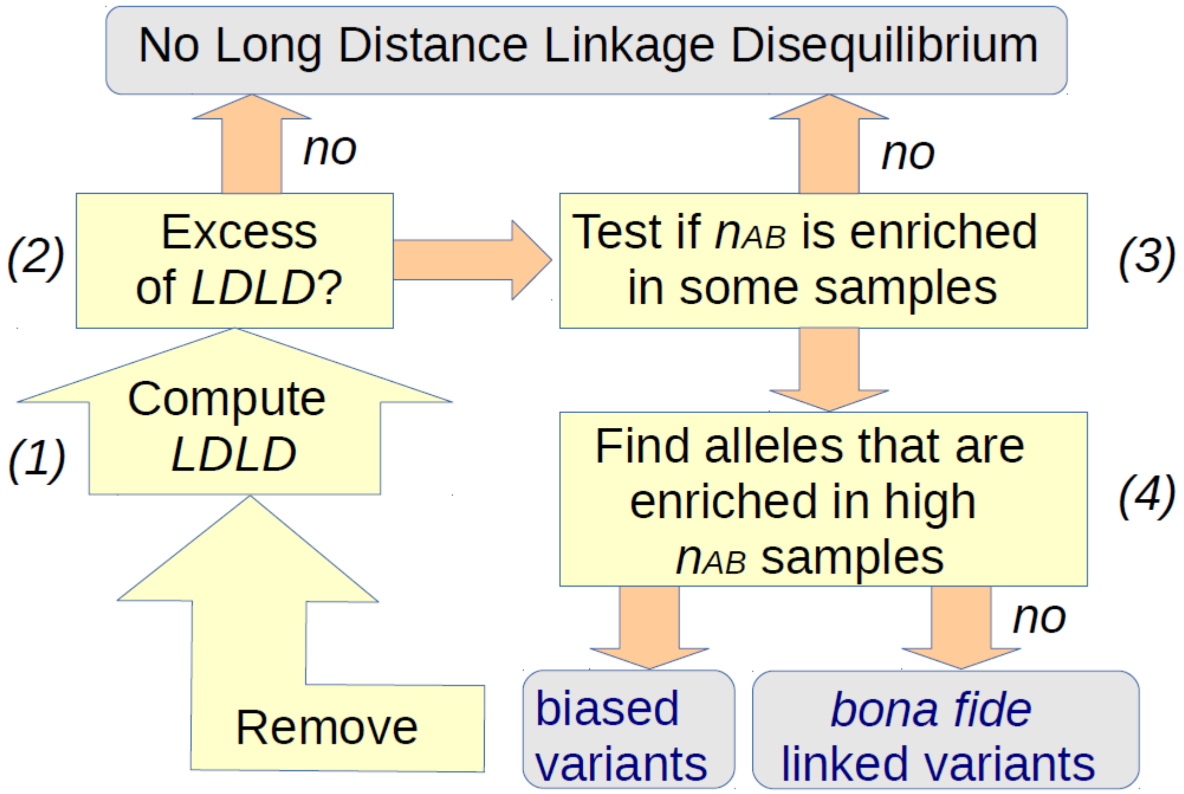

Supplementary Figure 1: Overview of the method. The pipeline consists of four main steps: (1) Linkage disequilibrium is computed across pairs of variants; (2) the number of linked variants is compared to an empirical null distribution, in order to determine whether in the dataset there is an excess of highly linked variants; (3) the number of minor alleles occurring in linked pairs is used to compute for each individual the contribution to the linkage signal. The variance in contribution across individuals is then compared to that of the empirical null distribution (by sampling an identical number of linked pairs, starting from the ones showing highest linkage in the reshuffled dataset) to verify whether certain individuals contribute disproportionately to the signal; (4) variants enriched in individuals contributing more to the linkage signal are labeled as potentially erroneous.

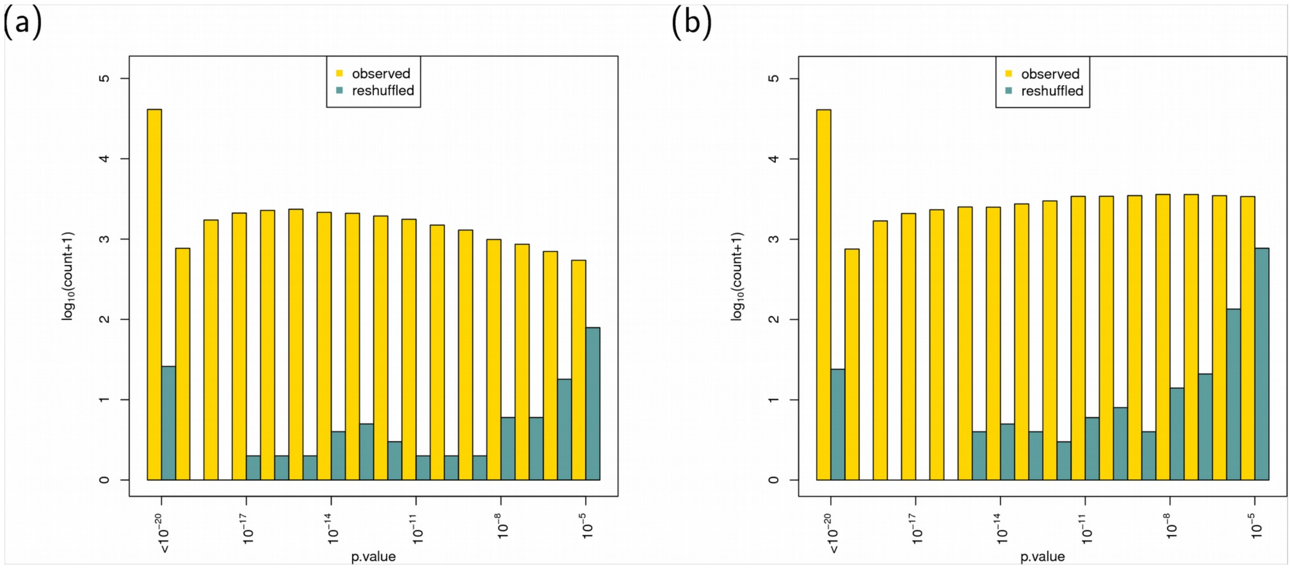

Supplementary Figure 2: Distribution of the observed (yellow) and permuted combined p-values (blue) when considering variants for which one or more populations have minor allele frequency at least 1% (a) or 5% (b), in the coding regions of the 1000 genomes dataset. Only positive linkage is considered here.

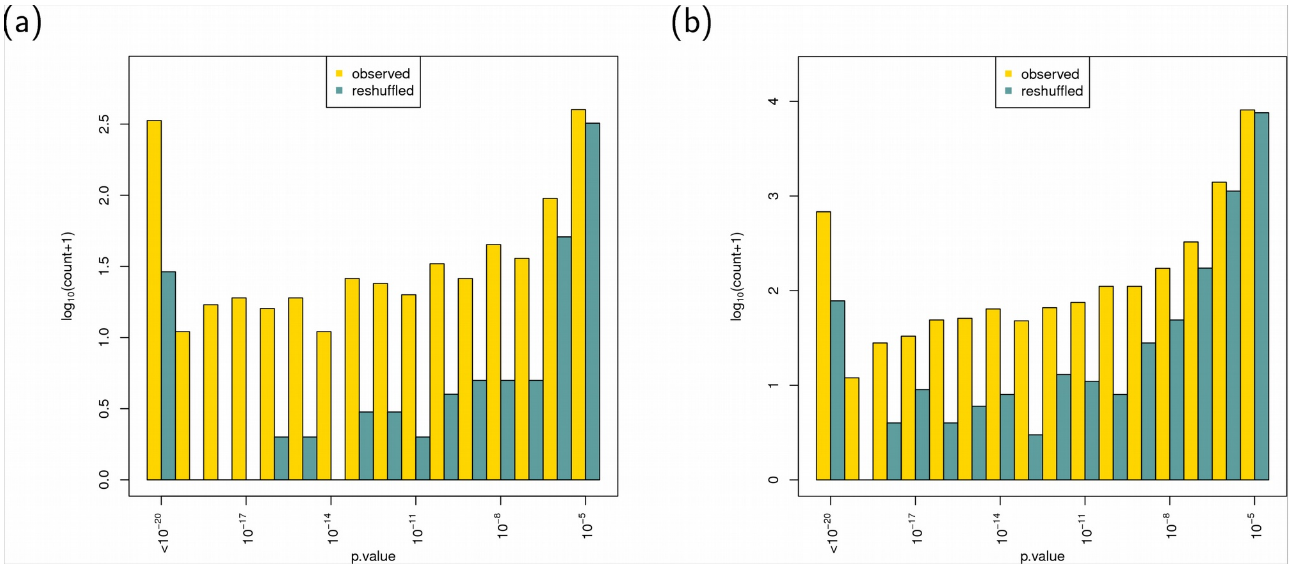

Supplementary Figure 3: Distribution of the observed (yellow) and permuted combined p-values (blue) when considering variants for which one or more populations have minor allele frequency at least 1% (a) or 5% (b), in intergenic regions of the 1000 genomes dataset. Intergenic variants were subsampled to match the number of variants present in coding regions. 10 different samples were used to compute the plots above. Only positive linkage is considered here.

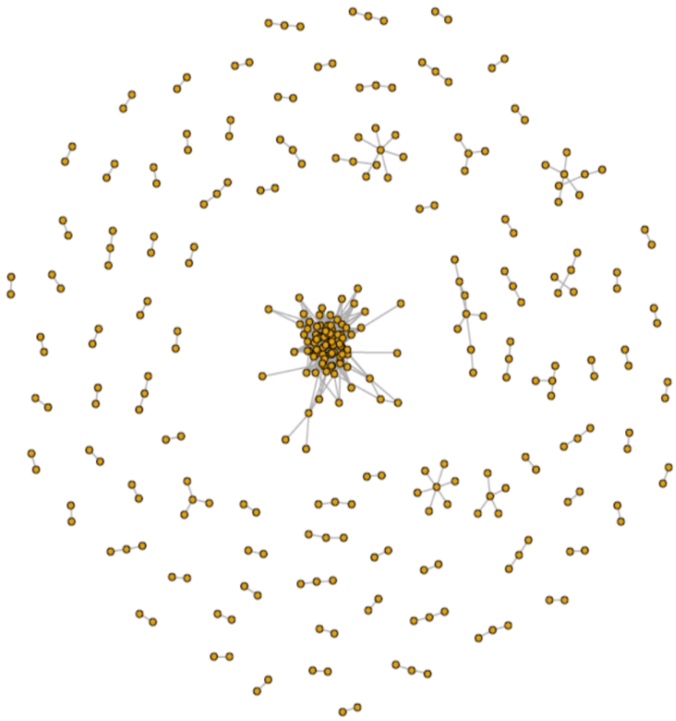

Supplementary Figure 4: Top 500 significant links (gray lines) between variants (dots) in the coding region of the 1000 genomes dataset.

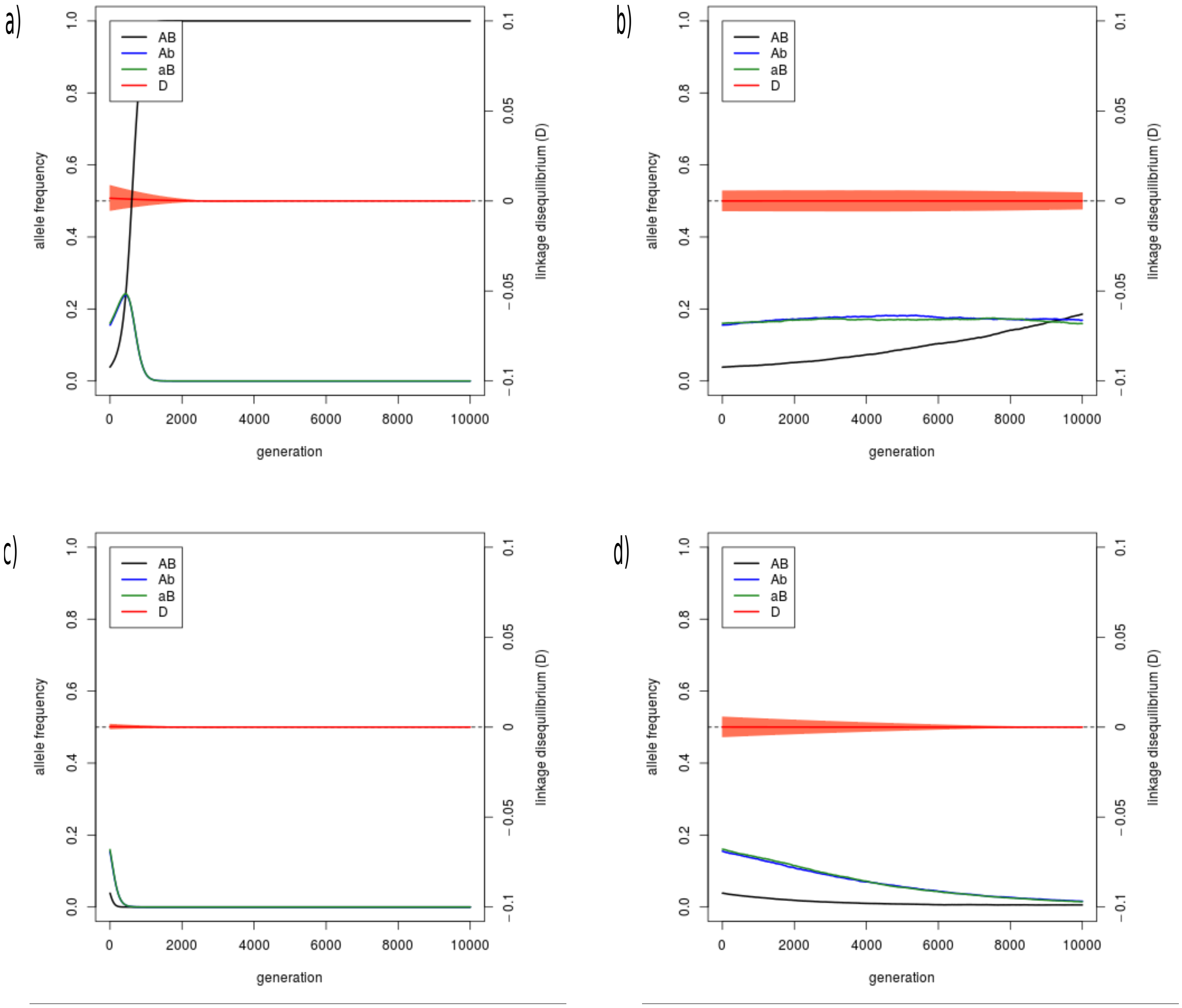

Supplementary Figure 5: Simulations for two alleles A and B with epistatic interactions. Gamete frequencies (solid lines) and linkage disequilibrium D (dashed line) for a population starting in linkage equilibrium with gametes A and B at allele frequency 20%. %. The shaded area includes the 95% confidence interval of D computed from simulations. a) Simulations of an advantageous combination of alleles A and B with strong selection (s=1%). A and B rapidly reach fixation. b) Simulations for an advantageous combination of alleles A and B with selection coefficients sampled from the estimated human distribution of fitness effects. A and B rapidly reach fixation. c) Simulations of antagonistic mutations (Ab and aB) with strong selection (s=1%). A and B rapidly reach fixation. d) Simulations antagonistic mutations (Ab and aB) with selection coefficients sampled from the estimated human distribution of fitness effects [3].

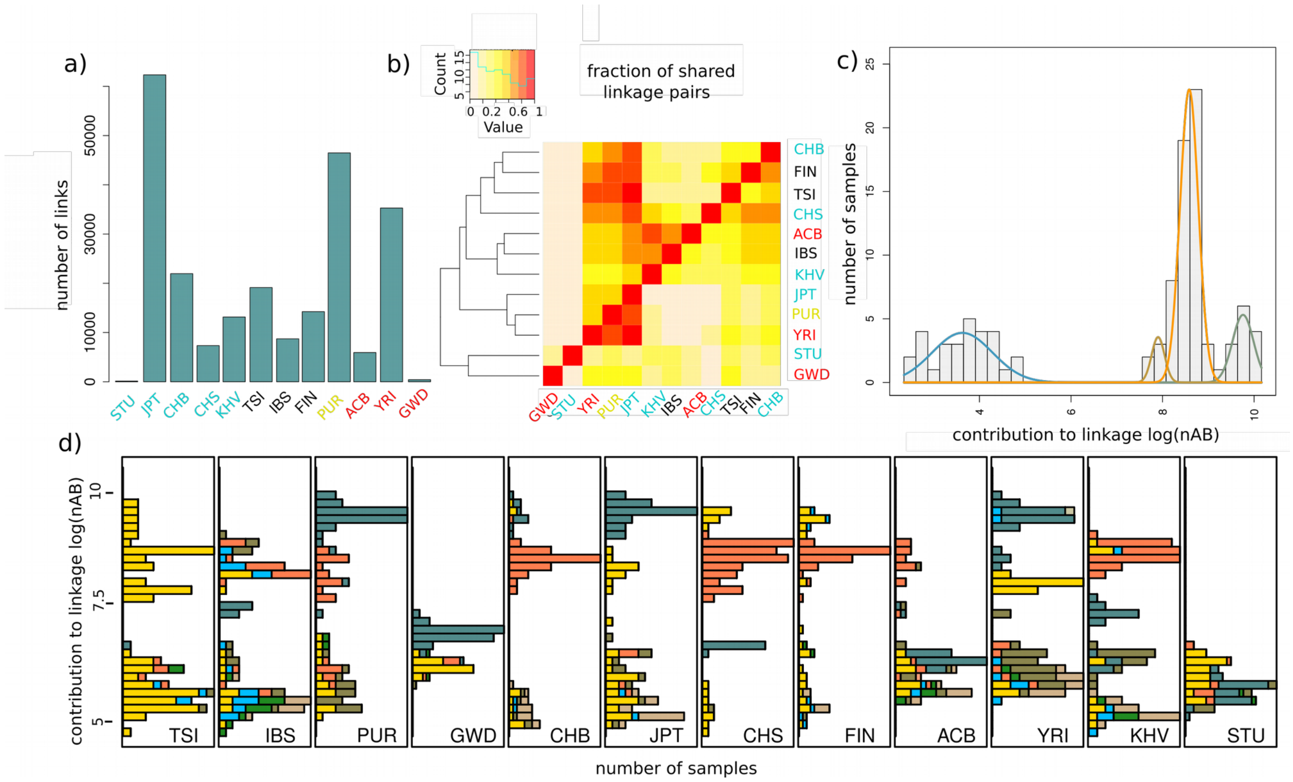

Supplementary Figure 6: Characteristics of inter-chromosomal linkage between coding region variants with minor allele frequency higher than 1%. a) Number of inter-chromosomal linked pairs of variants with a false discovery rate (FDR) < 5 % in coding regions of the 1000 Genomes populations. The FDR was calculated by comparing the p-value of each linked pair to the distribution of p-values after permuting chromosomes across individuals. Populations labels are colored according to the continent: blue for Asia, red for Africa, black for Europe and yellow for others. b) Fraction of inter-chromosomal linked pairs in one population (row) that are also linked in another population (column). c) Contribution of Chinese from Bejing individuals to the linkage signal (bars) in exomes given by the number of linked minor alleles (nAB). Individuals with similar nAB values were grouped by a Gaussian mixture model, whose fitted distributions are shown as colored lines. d) Distribution of nAB for individuals from different 1000 Genomes populations. Distributions are normalized for each population separately. Colors indicate the sequencing center per individual. Individuals sequenced in multiple centers were marked with a separate colors.

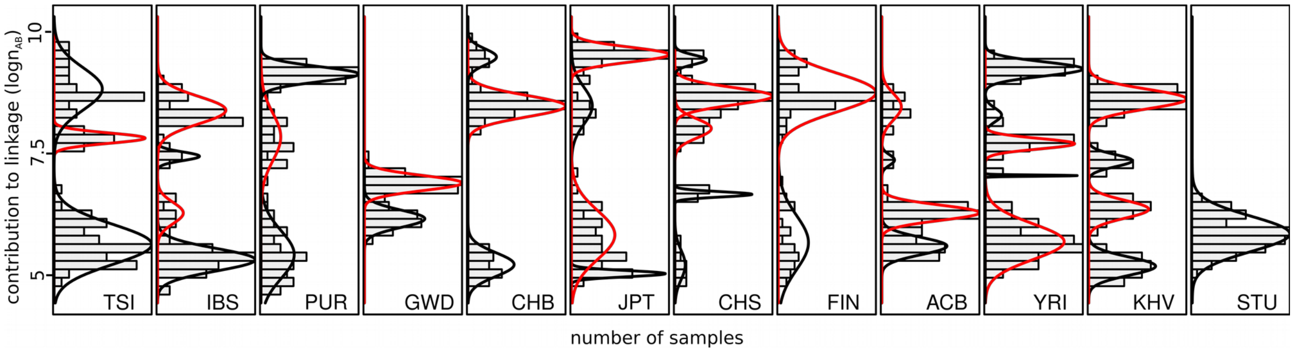

Supplementary Figure 7: nAB values (x-axis) in coding regions, for different populations of the 1000 Genomes Project and posterior Gaussian distributions underlying the different probabilities for the clusters identified by Mixtools (colored lines). Only positively linked variants with allele frequency >5% are considered here.

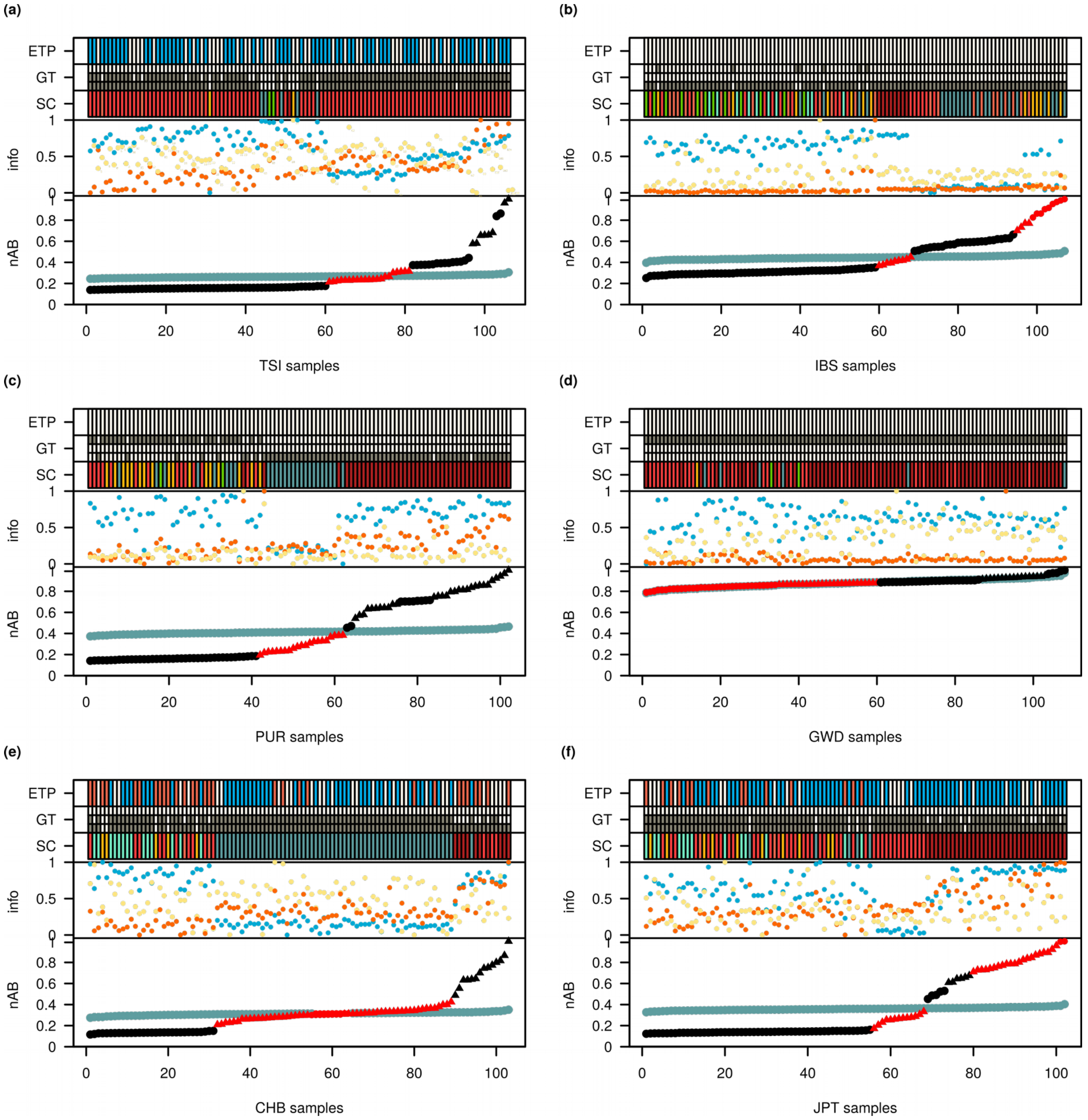

Supplementary Figure 8: nAB values (y-axis) for different populations of the 1000 Genomes dataset, when considering linkage between coding variants and minor allele frequency >5%. The x-axis ranks samples according to the observed contribution to the nAB value per sample, normalized by the maximum value per population. Blue dots indicate the empirical null distribution while black and red dots and triangles indicate the observed nAB. The different markers (triangles and dots) indicate clusters of samples with similar nAB identified by a Gaussian mixtures model (package Mixtools) while different colors indicate clusters of samples with similar log(nAB). The ”info field” represents the coverage on the exome (blue) and the total genome-wide sequence coverage (gold) and aligned coverage (orange) per sample, scaled between the minimum (shown as 0) and maximum (shown as 1) values per population. Different colors on the SC field indicate different sequencing centers. GT indicates different supporting chips platforms and ETP the specific platform used for the exome sequencing of the Phase I release of the 1000 Project, 454 (red) and Illumina

(blue). The populations represented are Tuscani (TSI), Iberians (IBS), Puerto Ricans (PUR), GWD (Gambians), Chinese from Bejing (CHB) and Japanese (JPT).

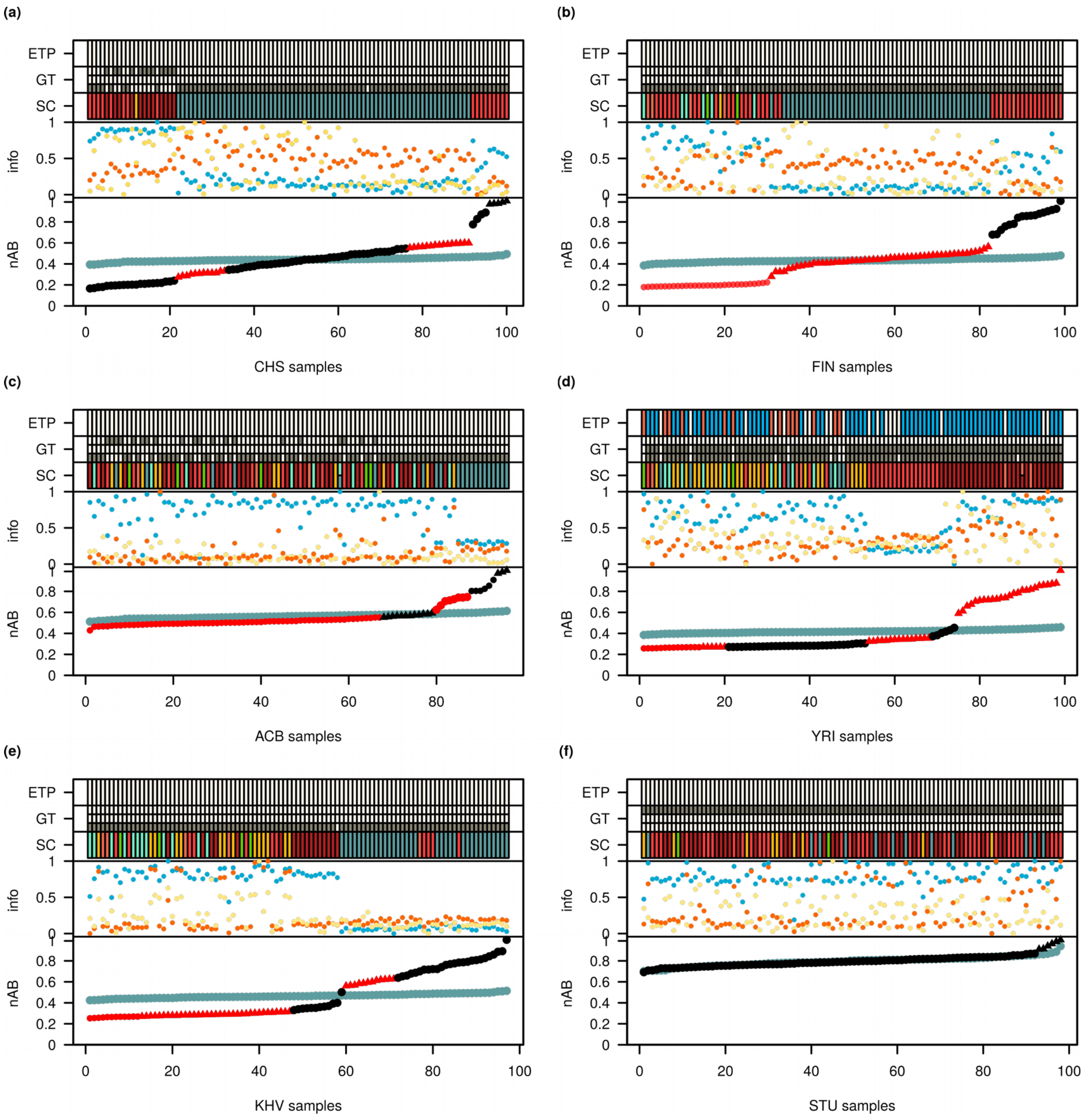

Supplementary Figure 9: nAB values (y-axis) for different populations of the 1000 Genomes dataset, when considering linkage between coding variants and minor allele frequency >5%. Labeling as in Supplementary Figure 8. The populations represented are South Chinese (CHS), Finnish (FIN), ACB (Caribbeans of African Ancestry), Yorubas (YRI), Vietnamese (KHV) and Tamil (STU).

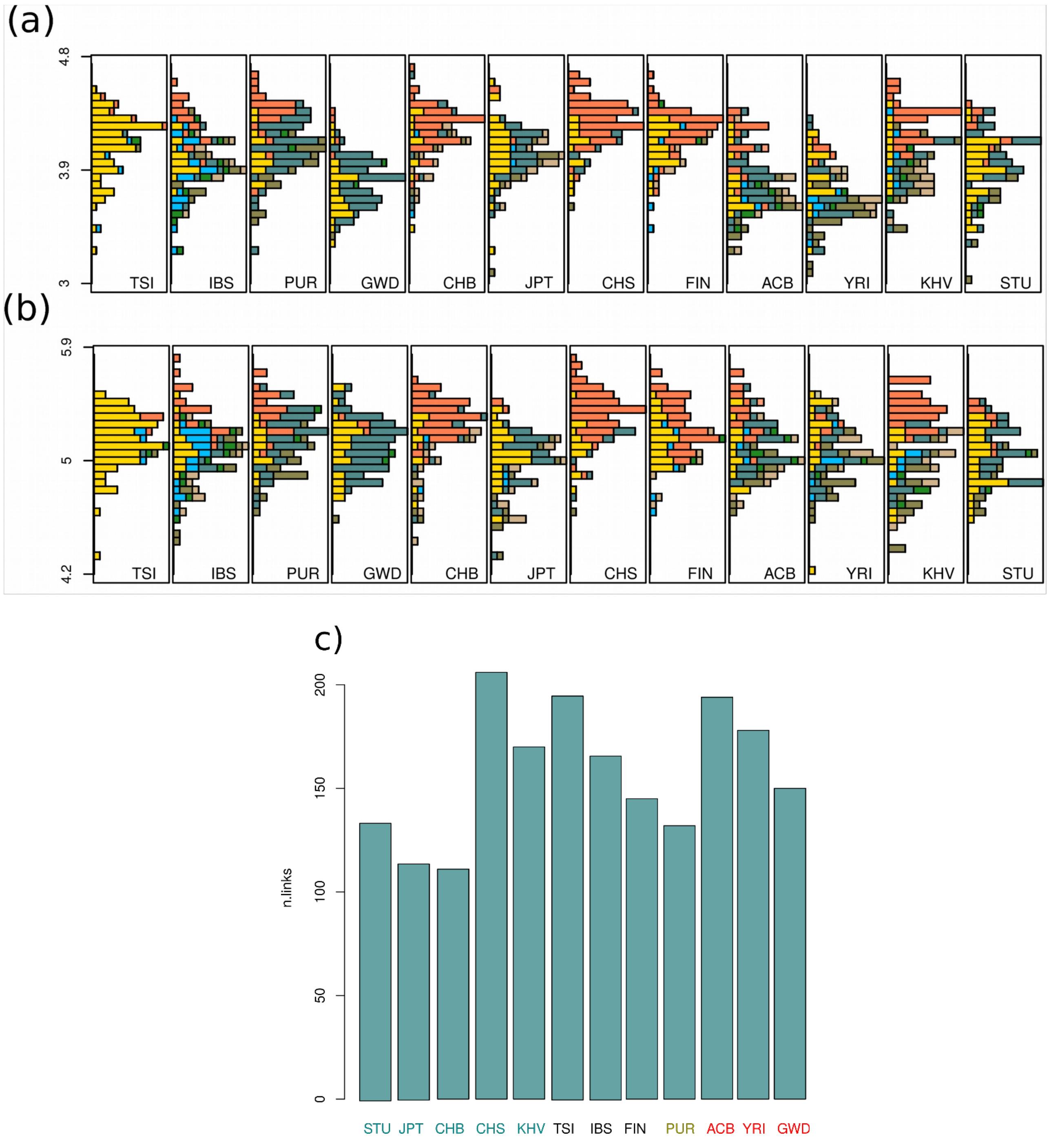

Supplementary Figure 10: Linkage between intergenic variants. (a-b) Sequencing centers and distribution of nAB for intergenic variants, when considering populations with minor allele frequency at least 1% (a) or 5% (b), in intergenic regions of the 1000 genomes dataset. Distributions are normalized for each population separately. Intergenic variants were as in Supplementary Figure 2. nAB was computed for variants with 20% and sequencing center information for intergenic variants subsampled following the scheme in to match the number of variants present in the coding regions of the 1000 genomes dataset. (c) Number of significant links per population identified in the intergenic dataset.

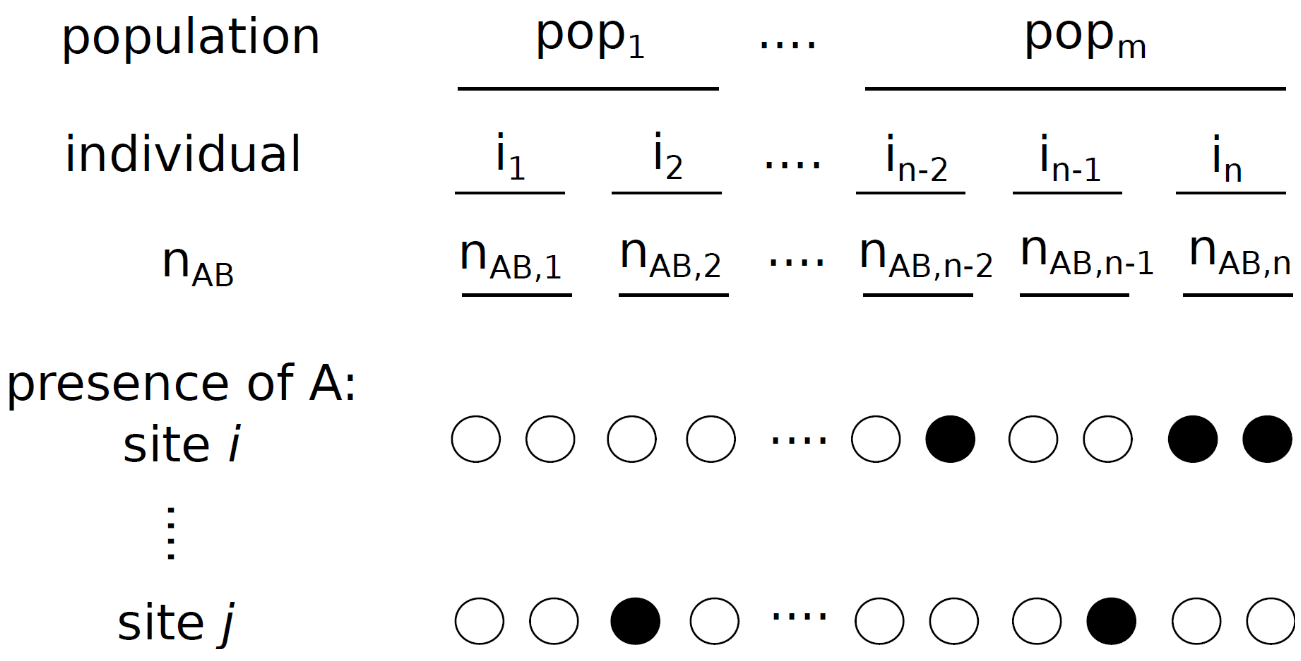

Supplementary Figure 11: Components of the Generalized Linear Mixed Model used to identify error candidates. The response variable is the absence (white) or presence (black) of each variants, in different individual chromosomes, here represented as circles. The predictors included in the null model (population+individual) and in the full model (population+individual+nAB) are shown above.

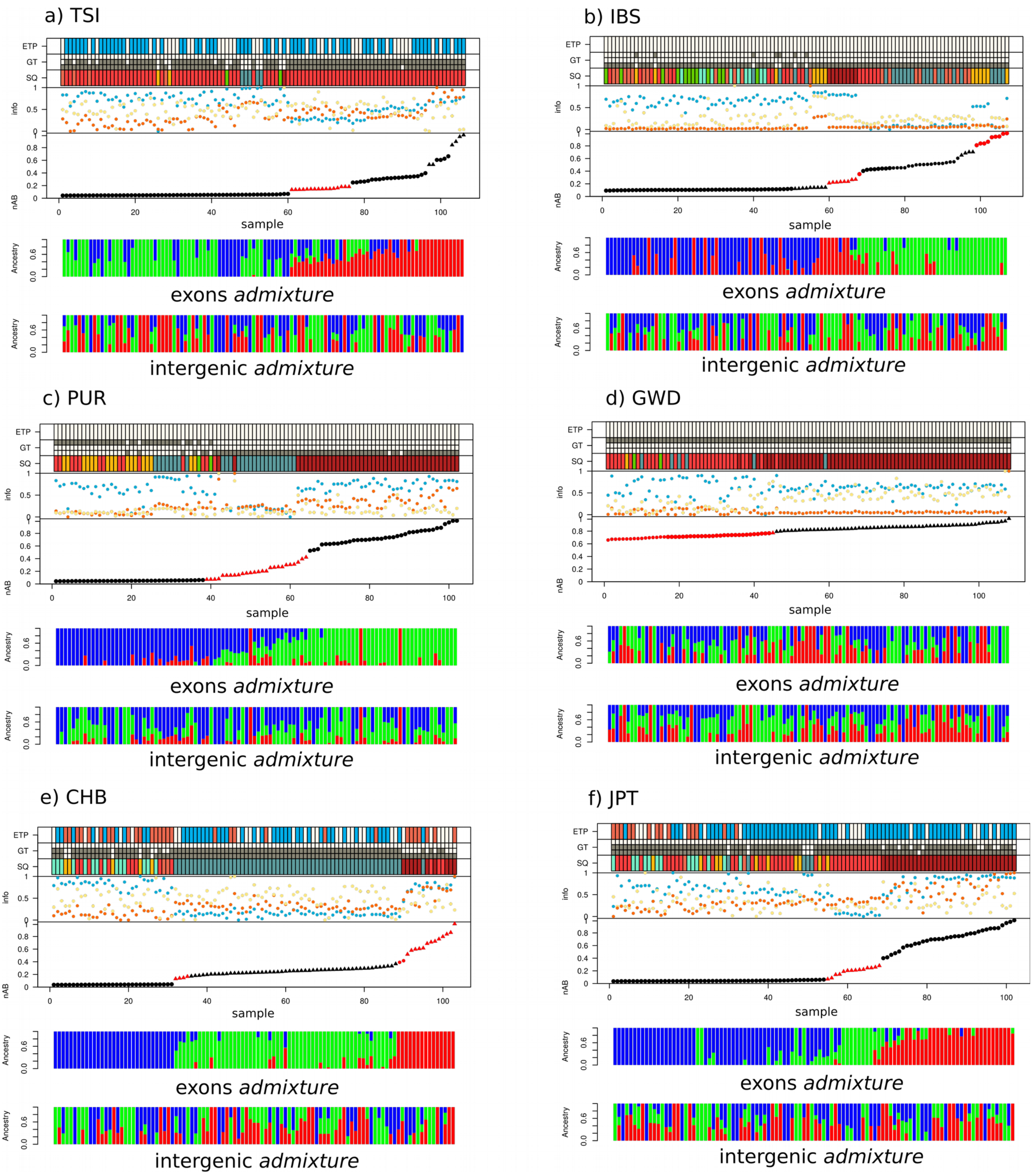

Supplementary Figure 12: nAB values (y-axis) for different populations of the 1000 genome dataset. The top panel follows the same description of Supplementary Figure 8. Below, the *Admixture* plots including 3 admixure components are reported for coding regions (above) and for an equal number of variants sampled in intergenic regions (below). The populations represented are Tuscani (TSI), Iberians (IBS), Puerto Ricans (PUR), GWD (Gambians), Chinese from Bejing (CHB) and Japanese (JPT). Only variants with minor allele frequency >1% are considered here.

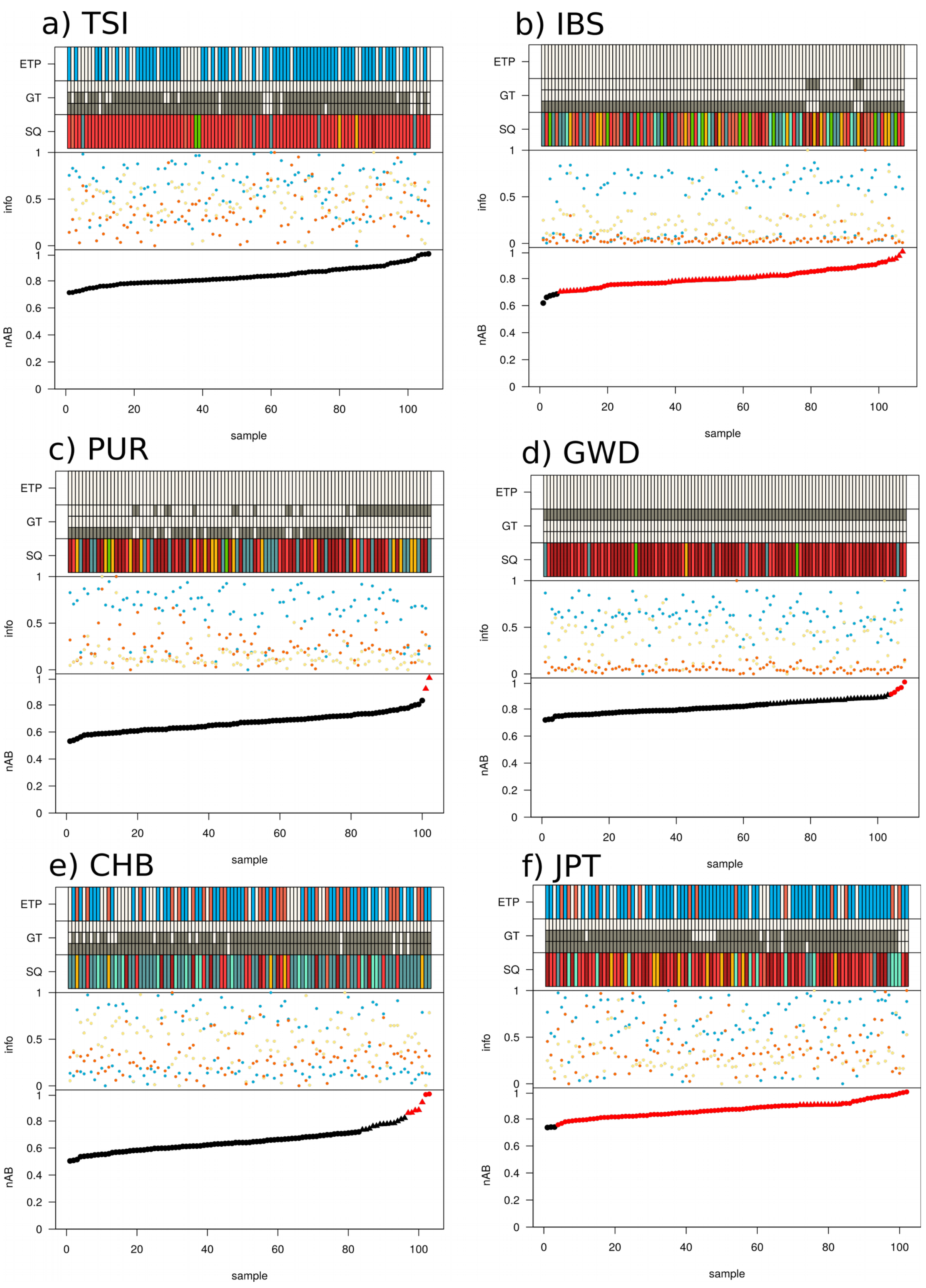

Supplementary Figure 13: nAB values (y-axis) for different populations of the 1000 genome dataset, when considering linkage between coding variants with minor allele frequency >5%, after the removal of potentially erroneous variants. Labeling as in Supplementary Figure 8.

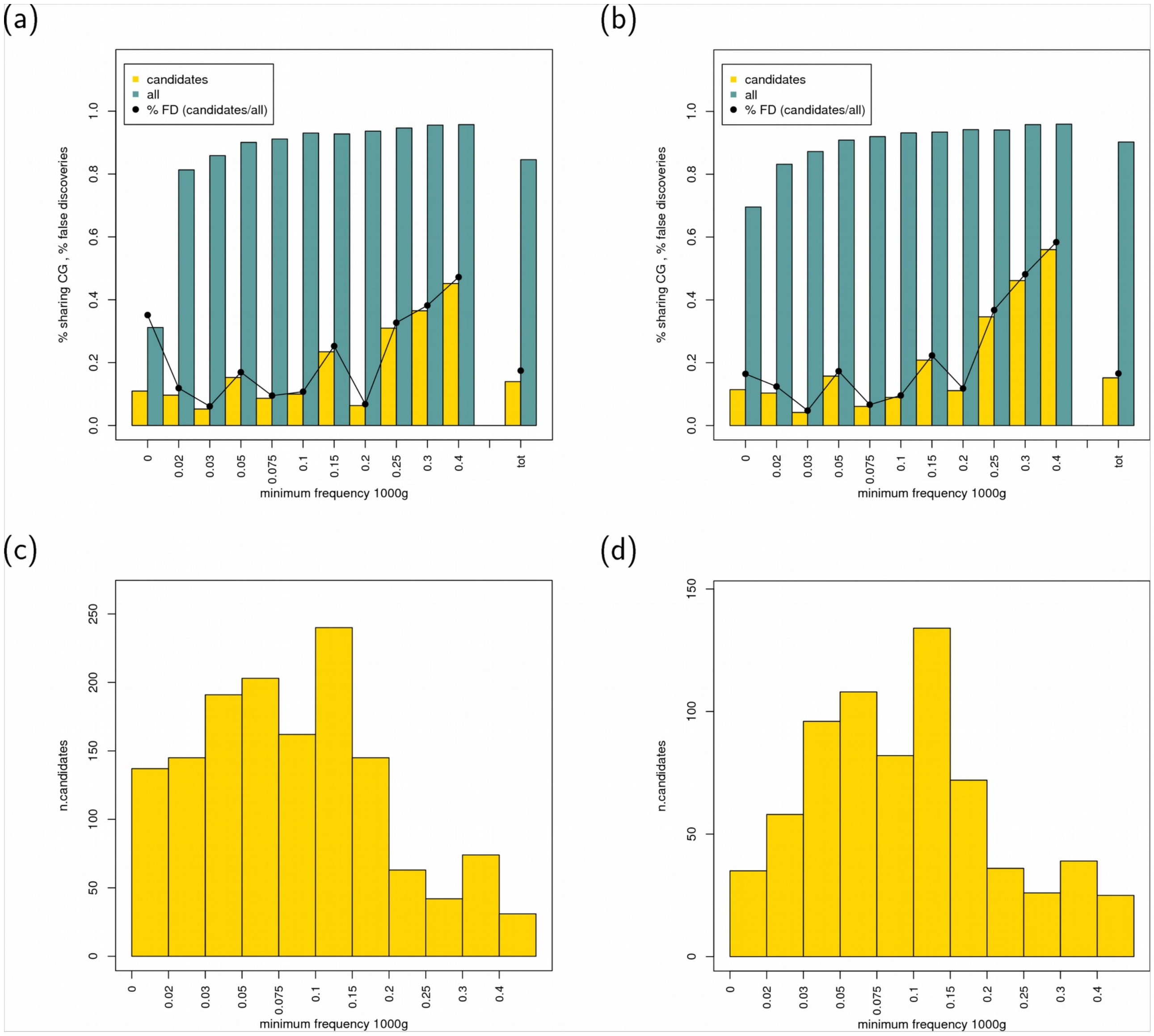

Supplementary Figure 14: Overlap of error candidates identified in the coding regions of the 1000 Genomes Project dataset with the Complete Genomics (CG) dataset. (a,c) and (b,d) correspond to variants reported in Supplementary Table 4 and 5, with a minor allele frequency threshold for the populations considered to compute nAB of 1% and 5%, respectively. (a,b) Bars indicate the fraction of variants observed in the CG dataset for candidates (yellow) and all variants (blue). Dots indicate the estimate empirical proportion of false positives among error candidates, calculated as the ratio between the variants present in Complete Genomics in the background set and among candidates. (c,d) Abundance of the candidates in different allele frequency bins, whose minimum frequency is shown on the left side (x-axis).

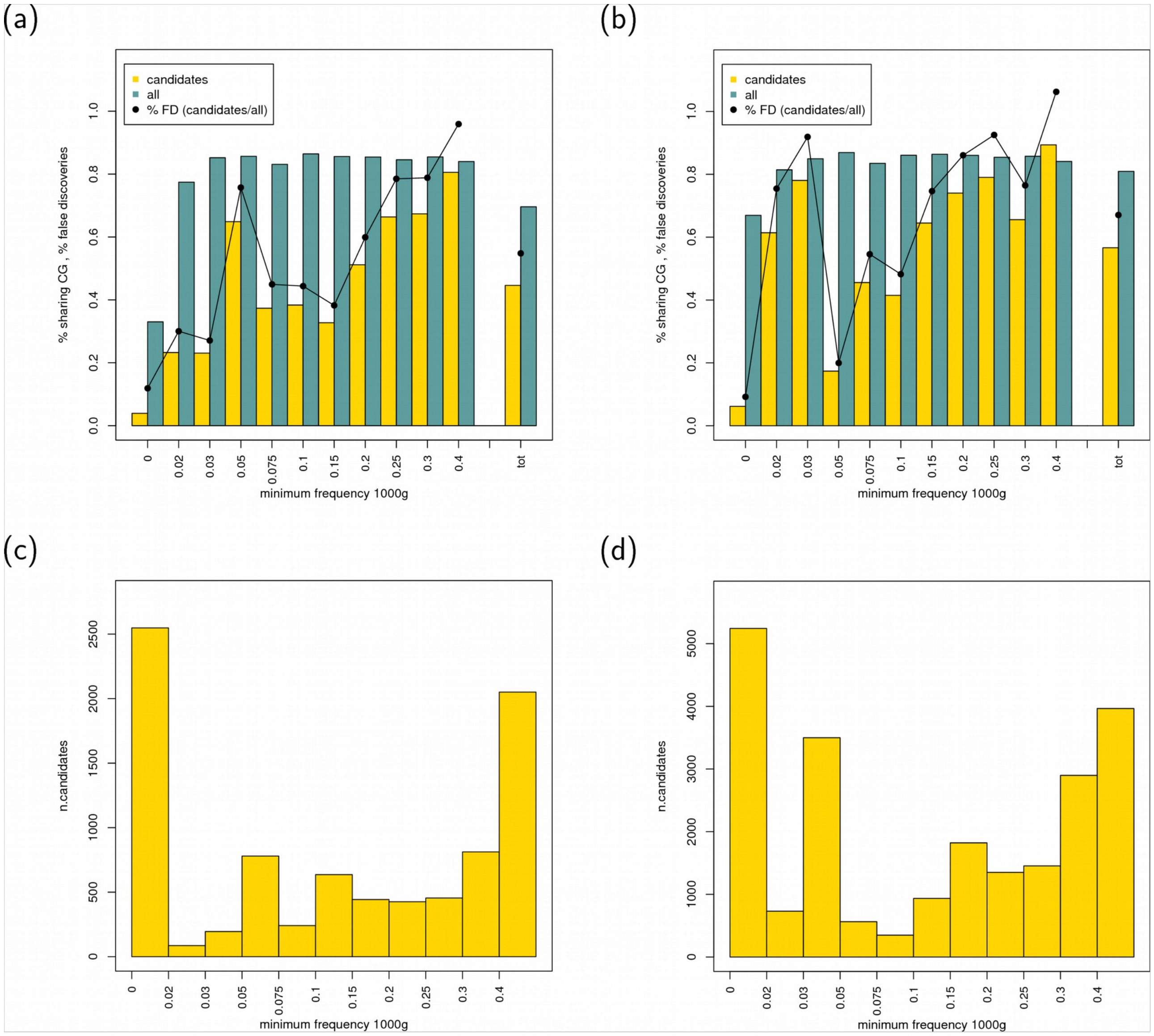

Supplementary Figure 15: Genome-wide overlap with the Complete Genomics (CG) dataset. Values of nAB used to predict errors are computed from linked intergenic variants reported in Supplementary Table 6 and 7. Minor allele frequency threshold equals 1% (a,c) and 5% (b,d). Description as in Supplementary Figure 14.

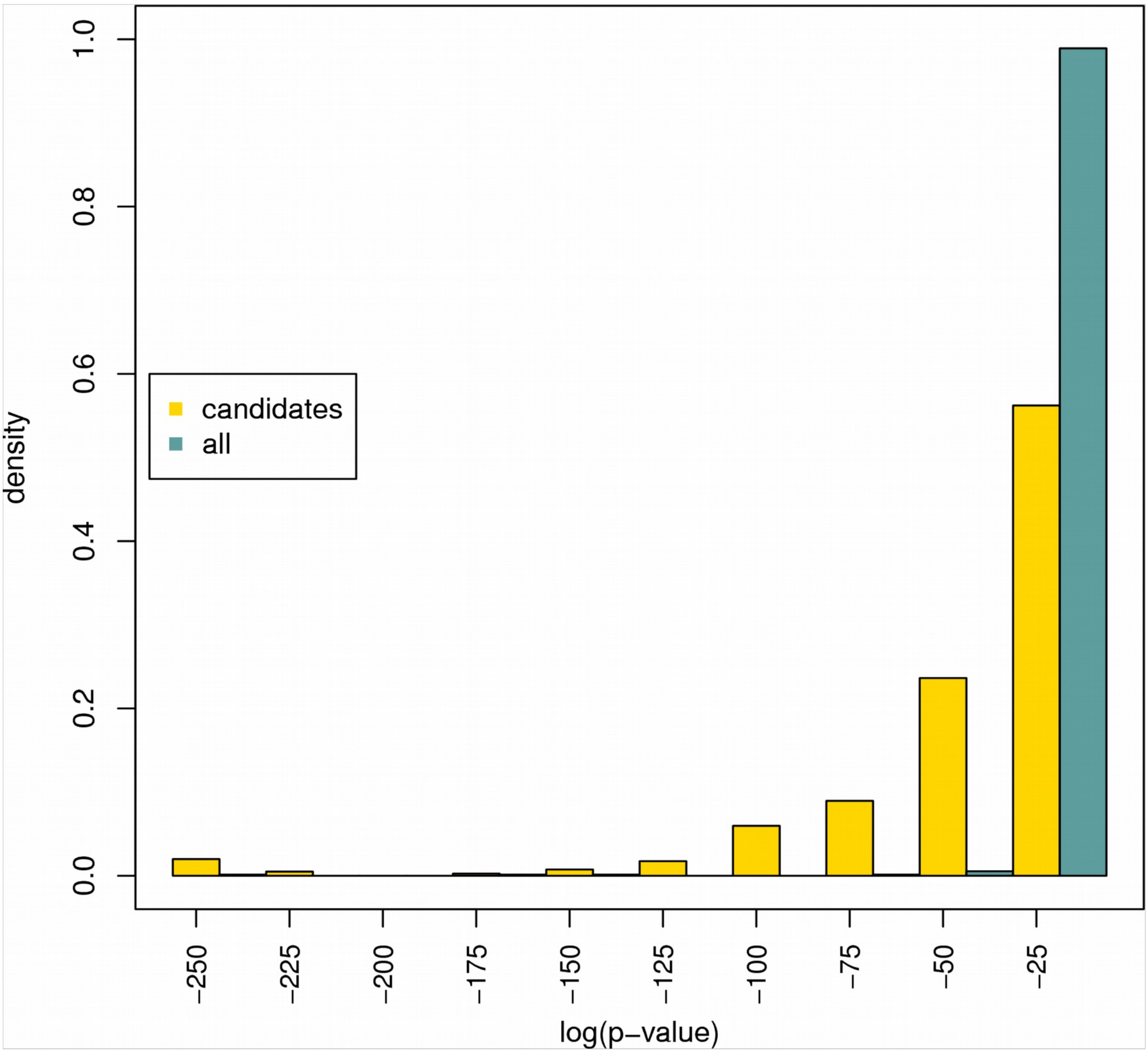

Supplementary Figure 16: Allele imbalance for errors candidates (yellow) and a background set of 10000 SNPs in the coding regions of the 1000 Genomes Dataset. Allele imbalance is computed by calculating the p-value of a binomial test with expectation 50% for the reads supporting the reference and the alternative allele at each site where at least one read supporting the alternative state is observed. The distribution of p-values is plotted in log scale.

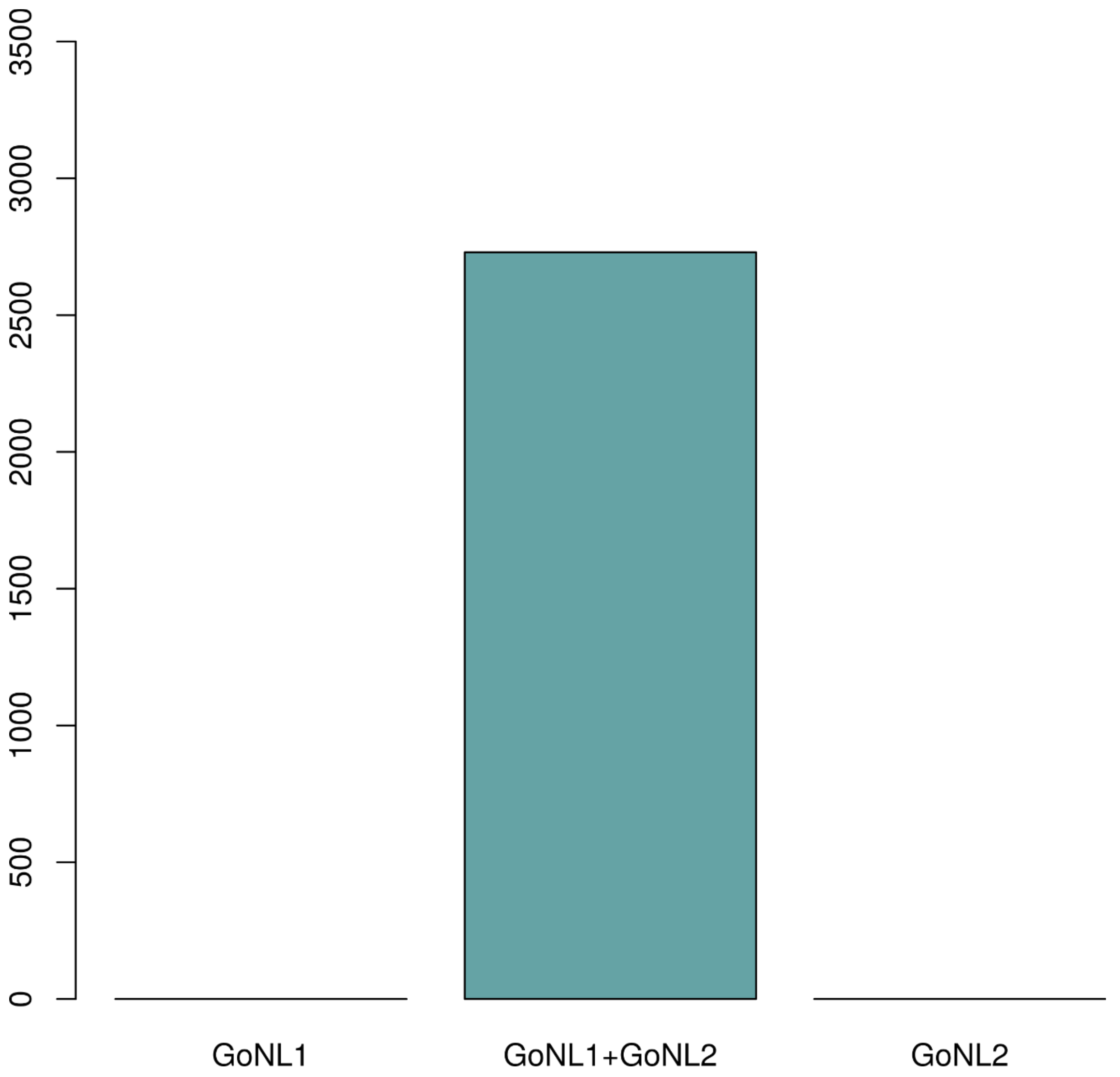

Supplementary Figure 17: Number of significant links between variants with frequency higher or equal than 5% in the coding regions of the GoNL1 and GoNL2 dataset. A merged vcf of the two datasets was created with vcf-tools.

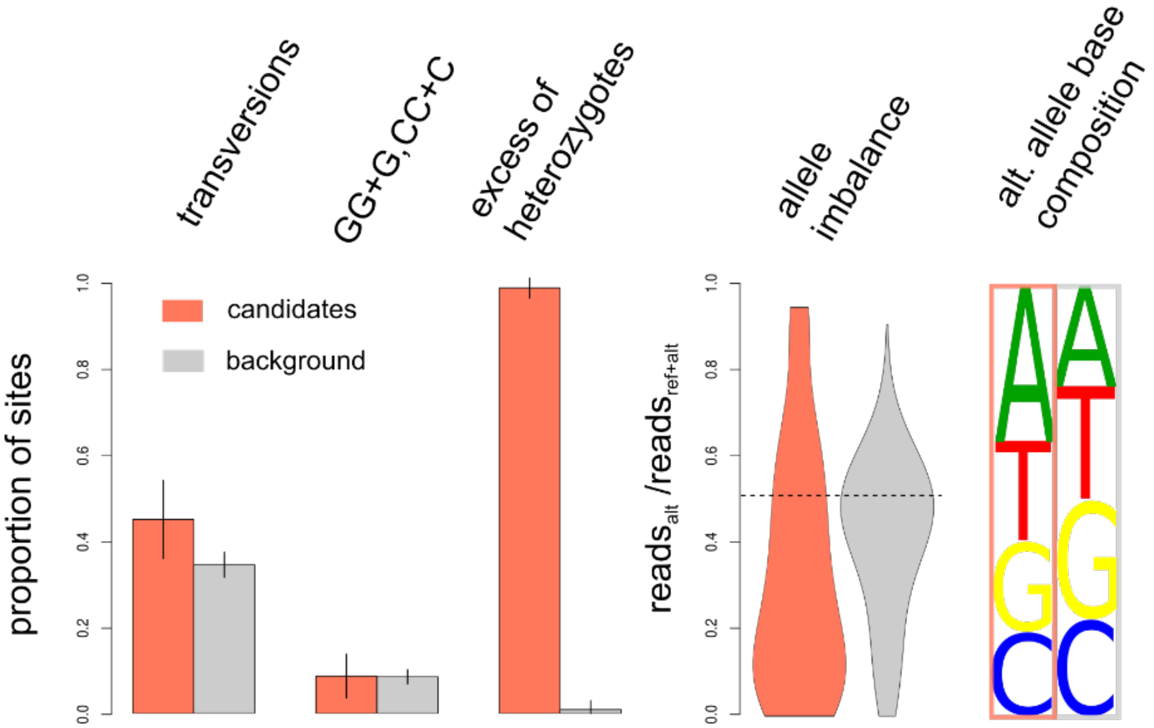

Supplementary Figure 18: Characteristics of error candidates in the GoNL dataset. For error candidates (red) and frequency-matched background variants (gray), the barplot shows the proportions of transversions versus transitions, alternative alleles introducing Gs or Cs after or before GG or CC dimers, and positions with significant excess of heterozygotes (pvalue<0.05). The violin plot shows the proportion of sequences supporting the alternative alleles in individual with at least one sequence showing the alternative allele. On the right, the base composition of alternative alleles is shown for error candidates (red) and background variants (gray).

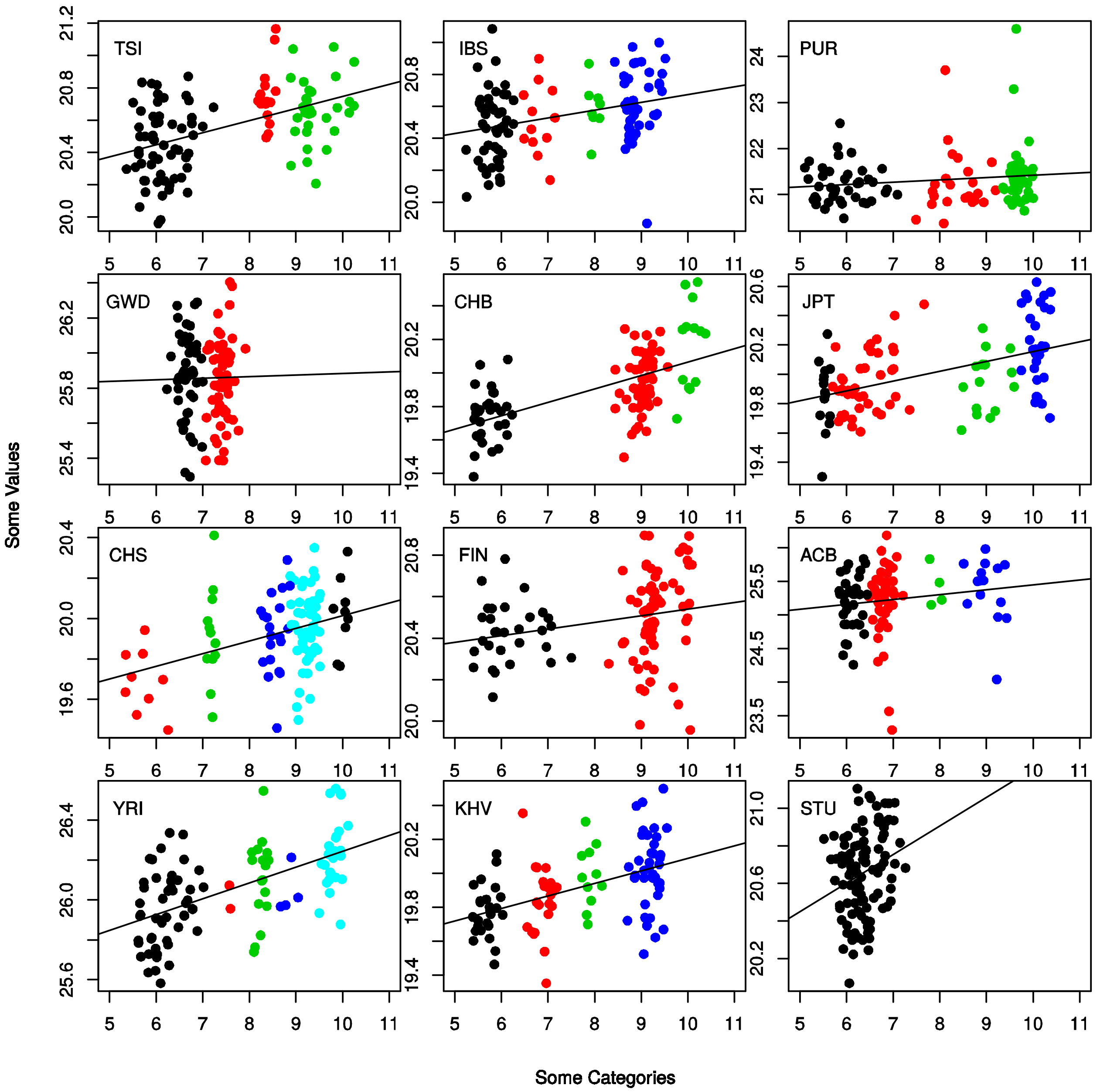

Supplementary Figure 19: Relationship between the contribution to the linkage signal across variants in the coding regions (log nAB for minor allele frequency>5%, x-axis) and the per individual count of minor alleles in the coding regions of the 1000 Genomes Project (number of mutation / 1000, y-axis). The 12 populations analyzed are reported separately in each panel. Batches identified by the Gaussian Mixture Model are shown in different random colors (note that the colors are independent in different populations). Regression lines between log nAB and the count of minor alleles are shown as black lines. Note however that samples are not independent from each other. To account for this non-independence, we tested the relationship between log(nAB) and the mutational load with a linear mixed model including the random categorical predictors Population and Batch, and their effects on the count of minor alleles. The categorical predictor Batch represents the sequencing batches identified by the Gaussian Mixture Model, each

shown with a different color. To estimate the effect of nAB we compared this model to a reduced model in which the effect of the predicor nAB is not present (p-value 0.000008954, likelihood ratio test). Note however that the size of this effect is estimated to be small (~0.2%), as the biggest variation in mutational load is explained by the predictor Population: the model that includes only the predictor Population explains already 98.9% of the variation in the data.

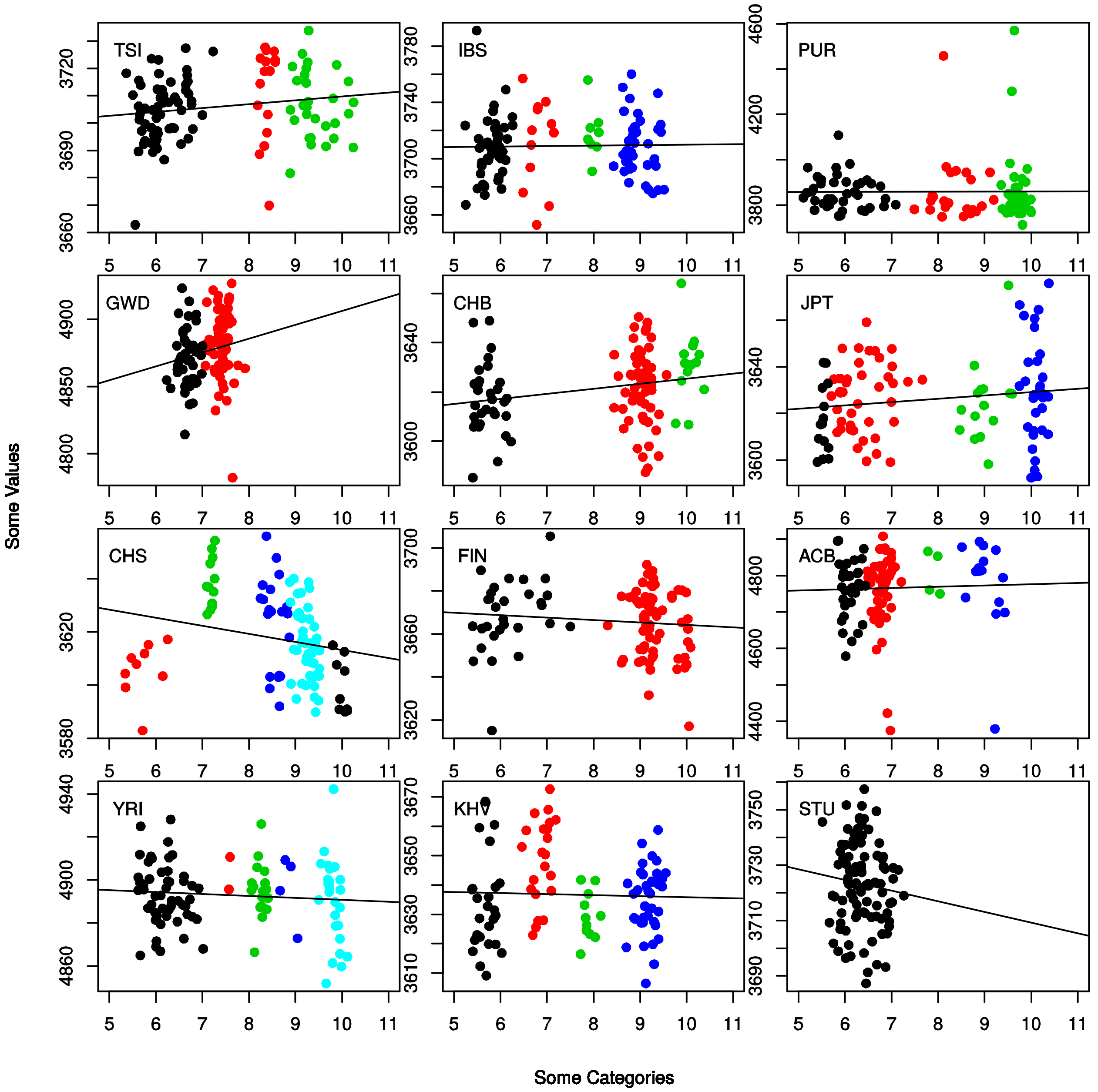

Supplementary Figure 20: Relationship between the contribution to the linkage signal across variants in the coding regions (log nAB for minor allele frequency>5%, x-axis) and the genome-wide count of minor alleles in individuals of the 1000 Genomes Project (number of mutation / 1000, y-axis). Description as in Supplementary Figure 21. The contribution to the linkage signal (lognAB) estimated from the coding regions does not exert a significant effect on the genome-wide mutational load (p.value 0.6394).

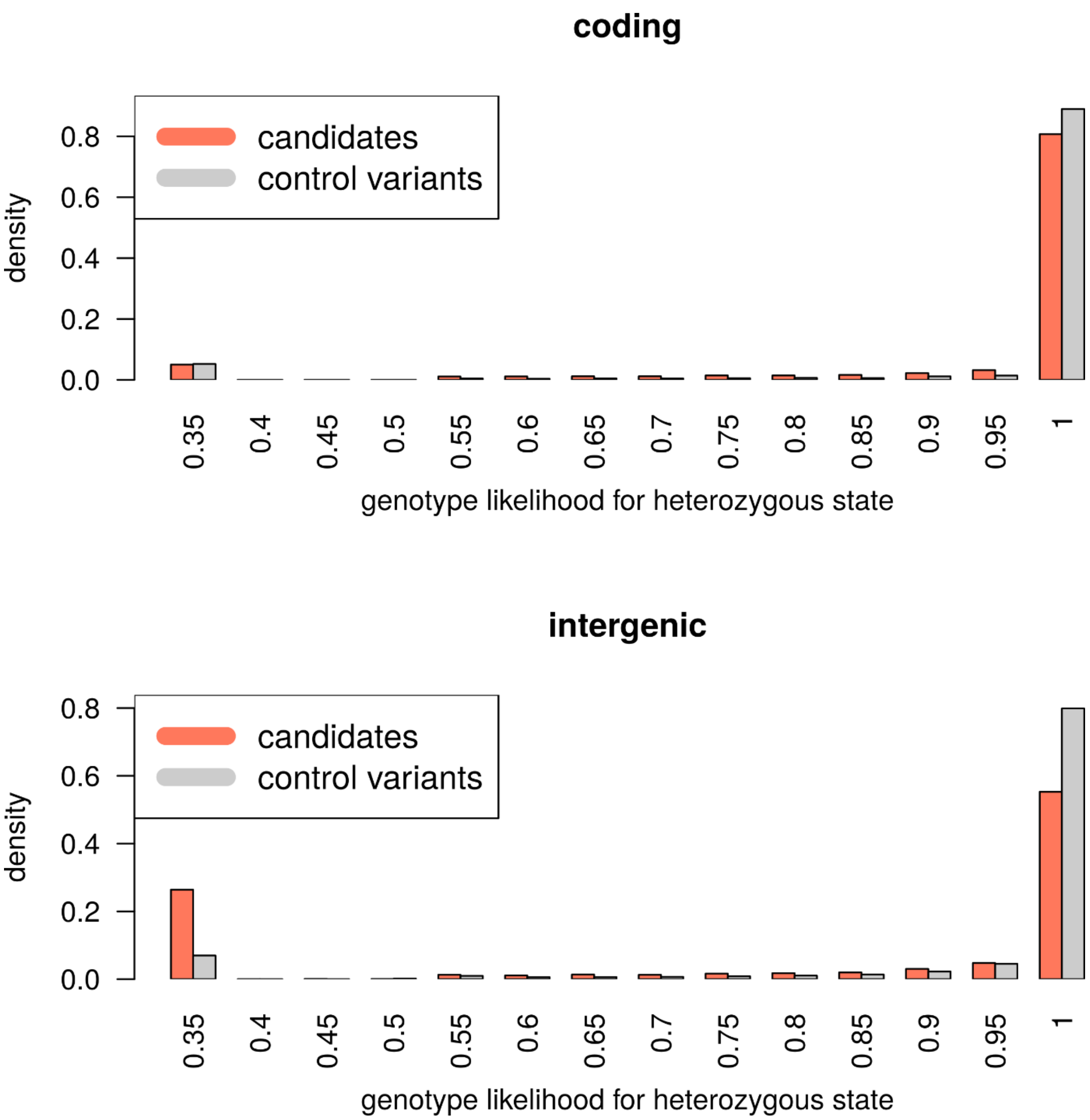

Supplementary Figure 21: Distribution of genotype likelihood for the heterozygous state in the 1000 Genome Project dataset. Only samples called as heterozygotes are here considered. Sites inferred as error candidates are reported in red and compared to other background variants, in gray, with MAF 5%. Error candidates estimated for coding regions and the intergenic dataset are reported in supplementary table 4 and 6, respectively.

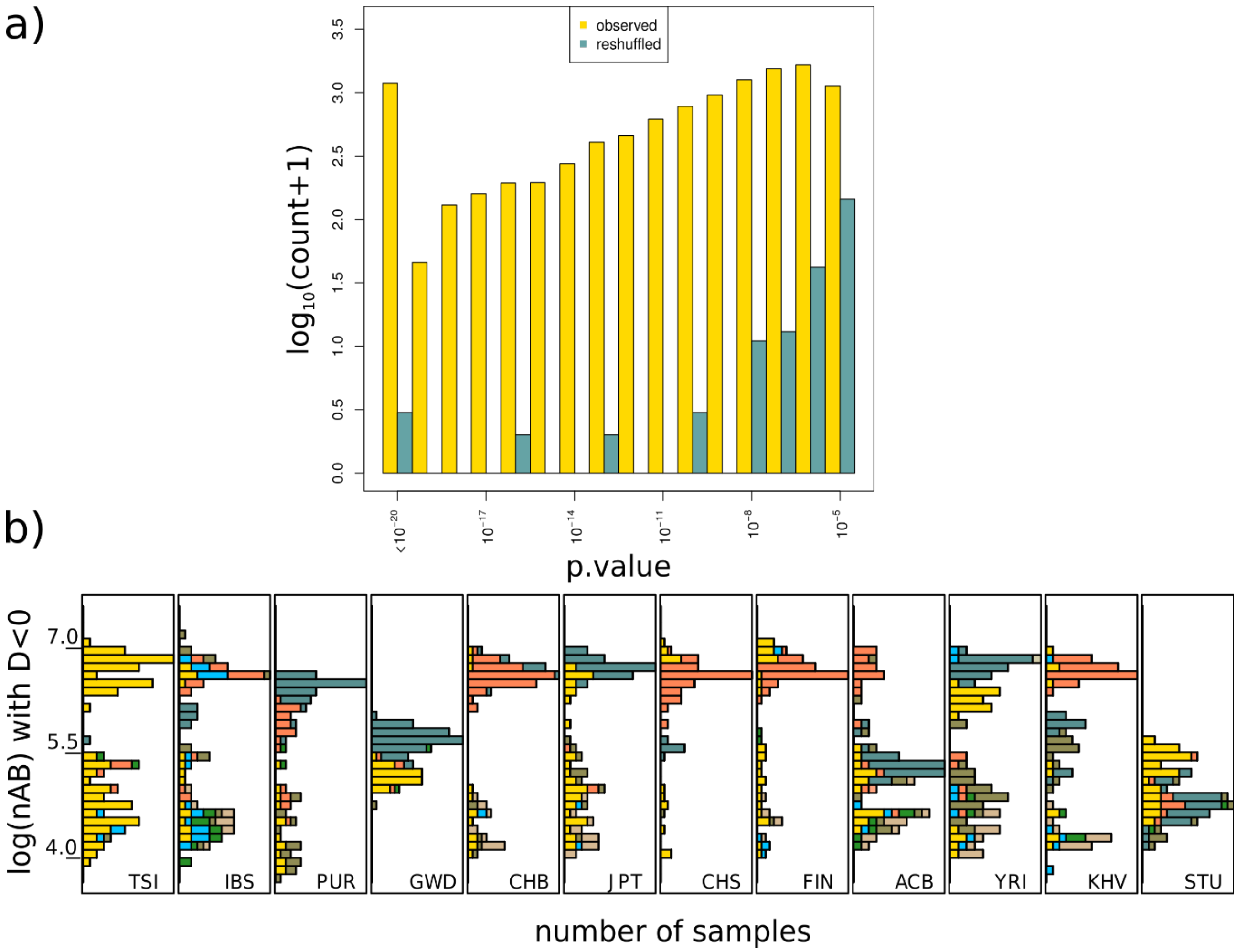

Supplementary Figure 22: Features of negatively linked variants (D<0). a) Distribution of the observed (yellow) and permuted combined p-values (blue) when considering populations with minor allele frequency at least or 5%, in the coding regions of the 1000 genomes dataset. Distributions are normalized for each population separately. Only the 1-tailed p-values of a negative association between minor variants are considered here. b) Distribution of nAB when considering only negatively linked variants in exomes for individuals from different 1000 Genomes populations. Colors indicate the sequencing center per individual. Individuals sequenced in multiple centers were marked with a different colors. Clear clusters of individuals sequenced in different sequencing centers can be identified on the basis of nAB when considering only minor variants linked with a linkage coefficient D<0 (corresponding to cases in which a minor and major variant are positively associated on two different chromosomes). Note however that the number of minor variants that are positively associated exceeds that of positively associated minor and major variants, indicating that errors with high allele frequencies are less abundant: the number of linked pairs of variants with FDR<0.05 that are negatively associated (11885) is 7.4 fold lower than that of positively associated ones (88268). This difference is even more extreme for the strongest associations, with a number of positively linked pairs larger than 10 fold over that of negatively linked pairs (lowest p.value bin

with p.value<10^-20, see (a) versus Supplementary Figure 2).

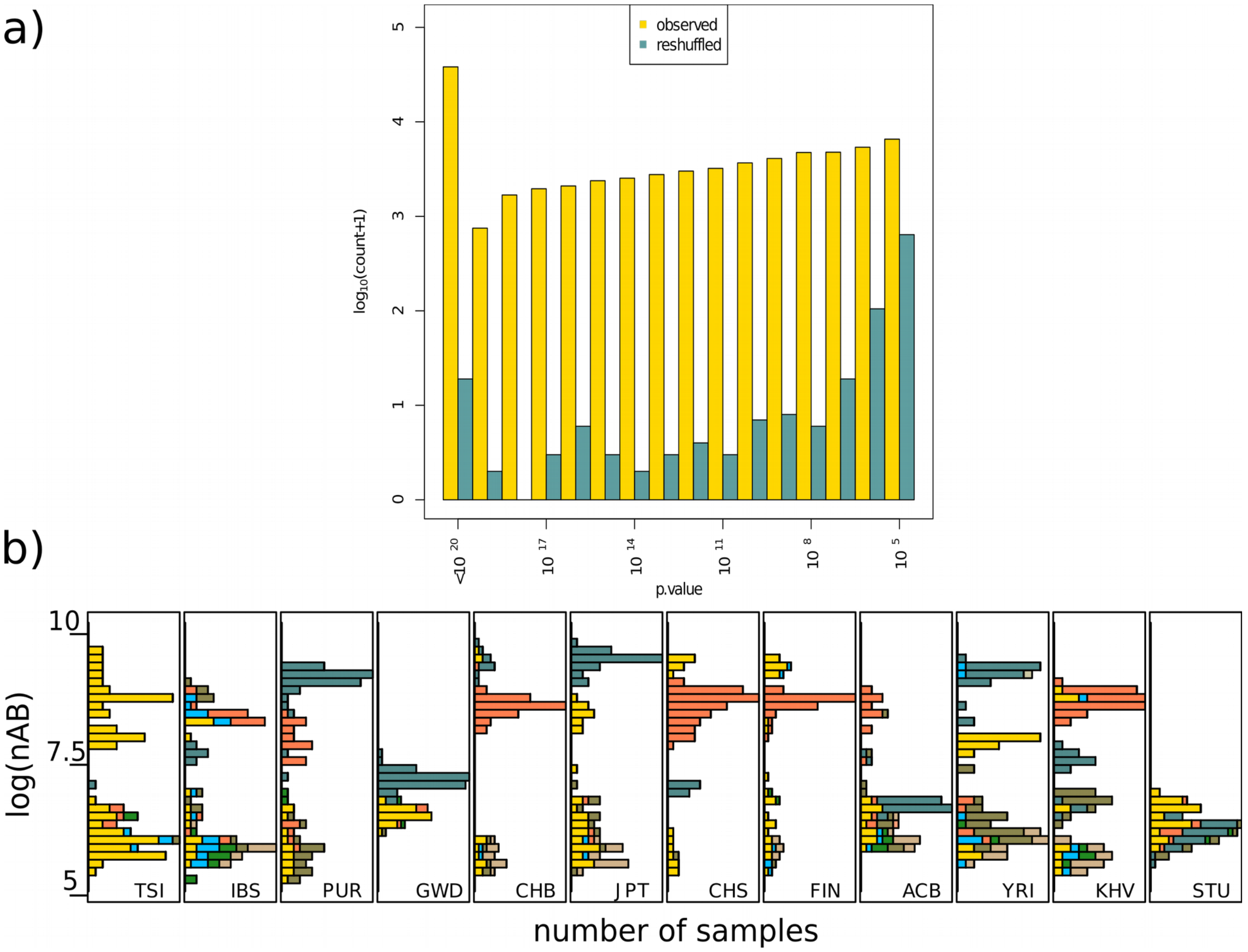

Supplementary Figure 23: Features of linked variants when both negative and positive linkage is considered. a) Distribution of the observed (yellow) and permuted combined p-values (blue) when considering populations with minor allele frequency at least or 5%, in the coding regions of the 1000 genomes dataset. 2-tailed p-values of a negative association between minor variants are considered here.

b) Distribution of nAB when considering both negatively and positively linked variants in exomes for individuals from different 1000 Genomes populations. Colors indicate the sequencing center per individual. Individuals sequenced in multiple centers were marked with a different colors. The number of significantly linked pairs with FDR<0.05 is 98662. Distributions are normalized for each population separately.

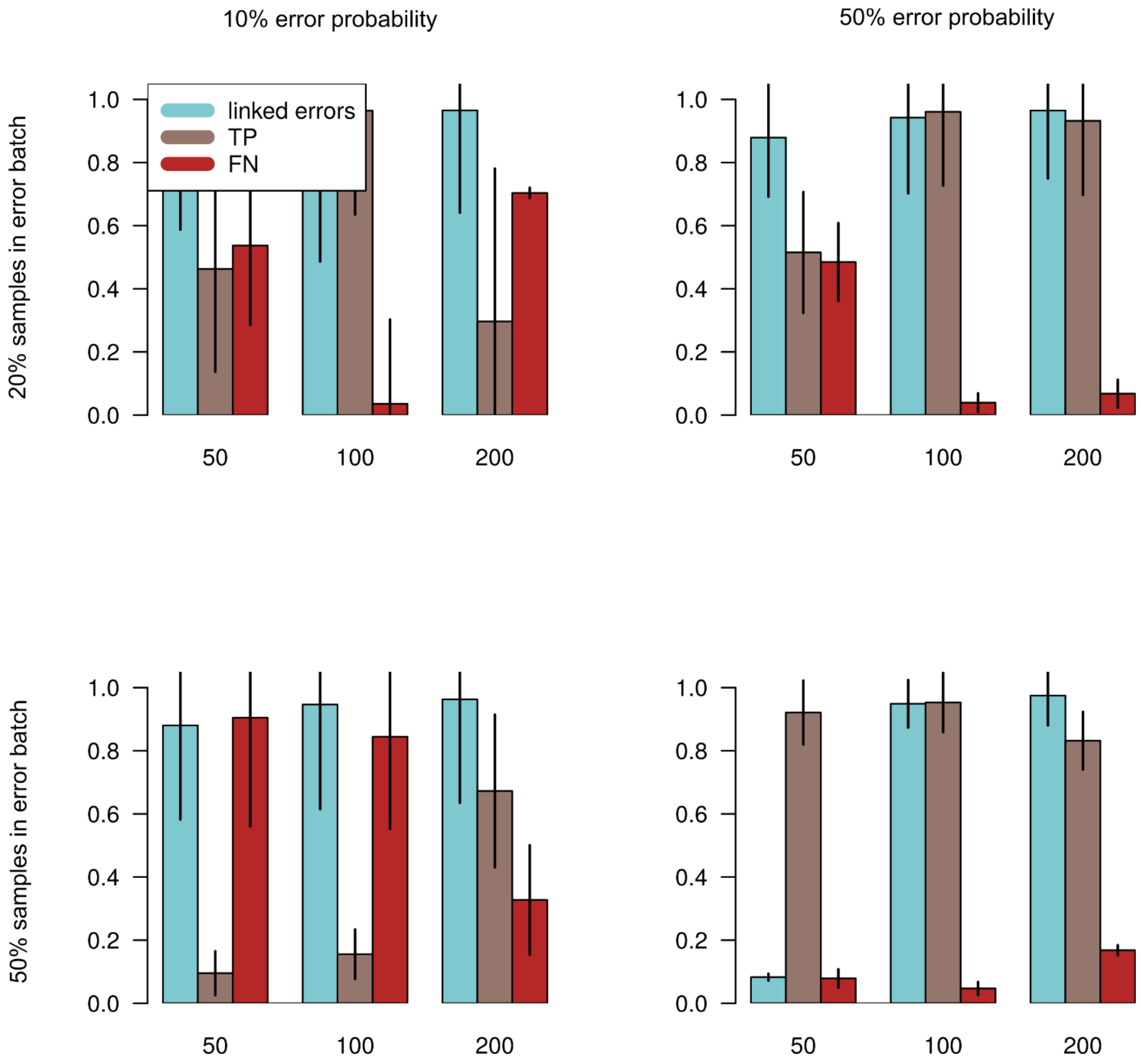

Supplementary Figure 24: Validation of the method for variants with minimum frequency 1%. We tested the pipeline on 5 simulated datasets with either 50, 100 or 200 unrelated individual genomes (x-axis). Individuals were divided into two batches, one with errors and encompassed either 20% (top) or 50% (bottom) of individuals. Errors were added to either 10% (left) or 50% (right) of the individuals. Errors were added in the form of false heterozygotes, leading to overall error rates equal between 10^{-5} (top left panel) and 0.000125 (bottom right). The plots show the proportion of significant links occurring between two errors (linked errors) (light-blue); the proportion of true positives, defined as the proportion of identified errors with frequency higher than 1% versus background variants; the proportion of false negatives, defined as the proportion of unidentified errors with frequency higher than 1%.

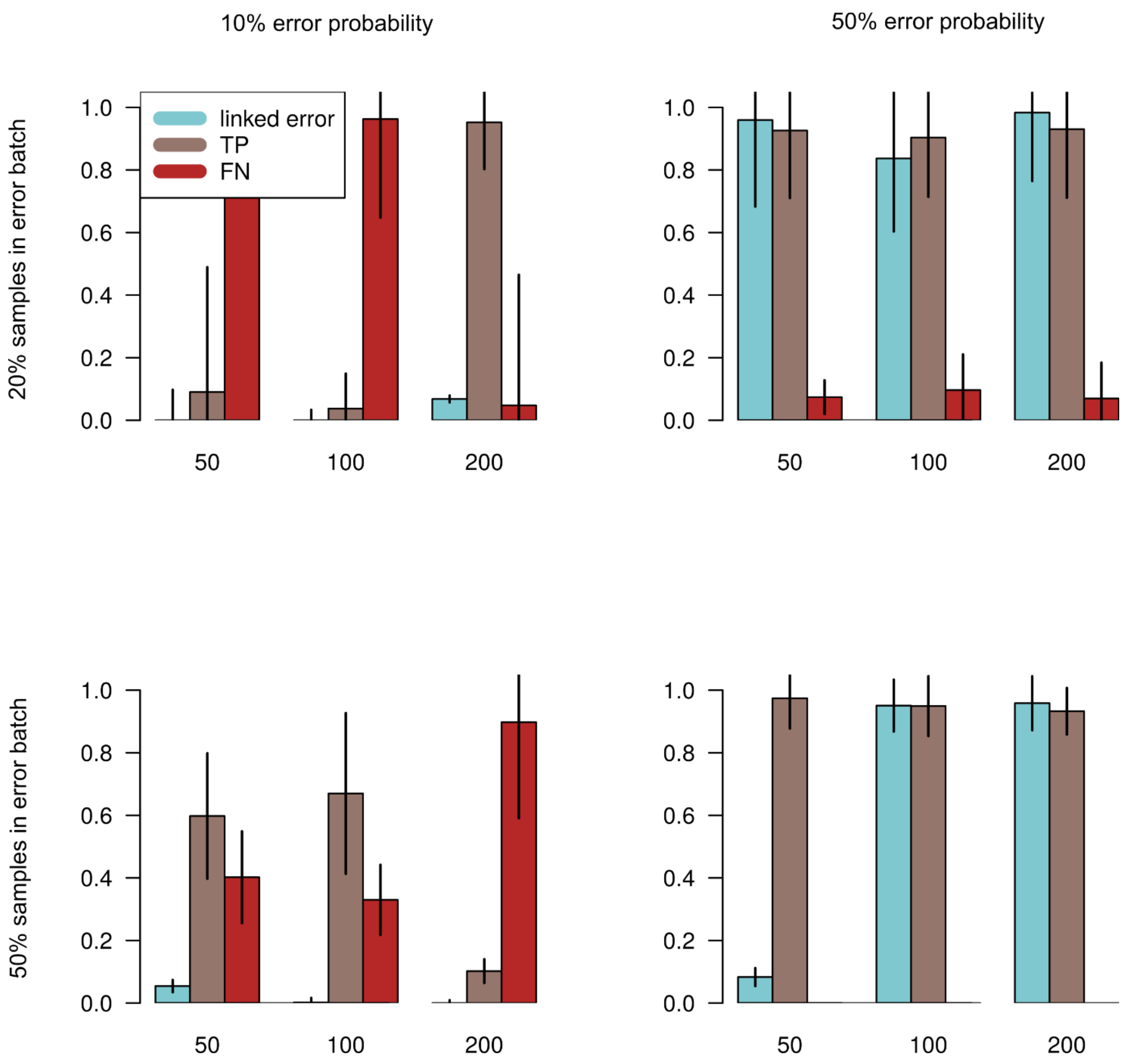

Supplementary Figure 25: Validation of the method for variants with minimum frequency 5%. Description as in Supplementary Figure 24. The plots show the proportion of significant links occurring between two errors (linked errors) (light-blue); the proportion of true positives, defined as the proportion of identified errors with frequency higher than 5% versus background variants; the proportion of false negatives, defined as the proportion of unidentified errors with frequency higher than 5%.

References

1. Weir, B.S., *Genetic Data Analysis II*. 1996.
2. Kulinskaya, E. and A. Lewin, *Testing for linkage and Hardy-Weinberg disequilibrium.* Annals of Human Genetics, 2009. **73**(2): p. 253-262.
3. Racimo, F. and J.G. Schraiber, *Approximation to the Distribution of Fitness Effects across Functional Categories in Human Segregating Polymorphisms.* PLOS Genetics, 2014. **10**(11): p. e1004697.
